# Platinum Complexes of Tetradentate *NCCN*-Coordinating Ligands: Structures and Photophysical Properties of Pt^II^, Pt_2_^III^ and Pt^IV^ Compounds

**DOI:** 10.1021/acs.inorgchem.5c04329

**Published:** 2025-11-07

**Authors:** Yana M. Dikova, Toby J. Blundell, J. A. Gareth Williams

**Affiliations:** Department of Chemistry, 3057 Durham University, Durham DH1 3LE, U.K.

## Abstract

Tetradentate proligands based on biphenyl appended at the 3 and 3′ positions with pyridin-2-yl rings (H_2_L^1^), quinoline-8-yl (H_2_L^3^) heterocycles, or one such pyridine and quinoline (H_2_L^2^), have been synthesized by palladium-catalyzed cross-coupling. These compounds were expected to undergo cyclometalation with K_2_PtCl_4_ to generate Pt­(II) complexes, PtL^1–3^, featuring *NCCN*-coordinated ligands. For H_2_L^2^ and H_2_L^3^, the corresponding Pt­(IV) compounds PtL^2–3^Cl_2_ were initially isolated, from which PtL^2–3^ could be obtained by reduction with zinc. In contrast, H_2_L^1^ gave an unusual Pt­(III) dimer, Pt_2_L^1^_2_Cl_2_, and subsequently PtL^1^ upon reduction. All of the complexes have been structurally characterized in the solid state by X-ray diffraction. The quinoline-containing complexes of L^2^ and L^3^ feature one or two 6-membered chelates, respectively, with an accompanying twisting of the quinoline ring(s) relative to the biphenyl unit to allow the metal ion to achieve its preferred square-planar coordination. PtL^1^ displays intense green phosphorescence in solution at room temperature with a quantum yield of 67%. The deep-red emission of PtL^2–3^ is weaker due to suppressed triplet radiative rate constants as well as faster nonradiative decay. The Pt­(IV) complexes emit only at 77 K, with long lifetimes of around 300 μs, while the Pt­(III) dimer shows no detectable emission.

## Graphical abstract

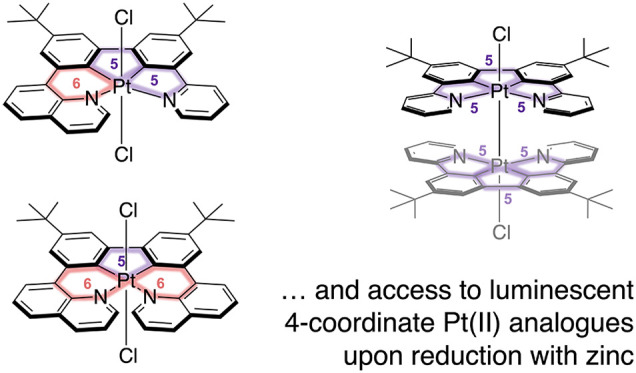

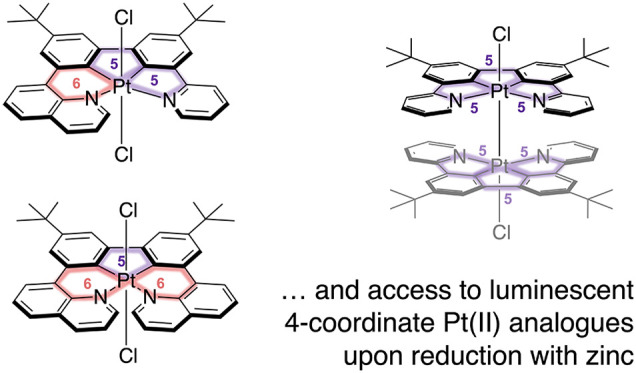

## 1 Introduction

The binding of aryl–heterocycle compounds to transition metal ions through cyclometalation—with concomitant formation of M–C and M–N bonds within a 5- or 6-membered chelate ring—is widely encountered.^1^ Cyclometalated complexes featuring such M­(*NC*) units have proved particularly valuable in the development of molecular phosphors—compounds that emit light efficiently from triplet excited states.^2^ Complexes based on *fac*-Ir­(*NC*-ppy)_3_ are widely used in organic light-emitting diodes (OLEDs), where they promote emission from otherwise wasted triplet excitons (ppyH = 2-phenylpyridine).^3^ There has also been a long-standing interest in related Pt­(II) complexes.^4^ The square-planar geometries favored by this d^8^ ion open up the possibility of face-to-face intermolecular interactions to form bimolecular species either in the ground state (dimers and higher aggregates) or in the excited state (excimers), which may emit at lower energy than the isolated molecules.^5^

Von Zelewsky and Balzani originally investigated *cis*-Pt­(*NC*)_2_ complexes around 40 years ago {where *NC* = ppy, thpy (2-thienylpyridyl), and bhq (benzo­[*h*]­quinolyl)}.^6^ They found that such complexes formed ^3^MLCT states that were emissive at 77 K, but the emission was quenched at room temperature, except for *cis*-Pt­(thpy)_2_. The inherent flexibility of Pt­(II) complexes featuring bidentate ligands, with respect to rotation of the planes of the ligands relative to one another (*i.e*., from square planar to D_2d_ symmetry), promotes nonradiative decay pathways via higher-lying d–d states.^7^ The use of more rigid tridentate^8^ or tetradentate^9^ ligands can mitigate this problem, leading to superior performance at room temperature. Accordingly, the concept of linking together two ppy-like *NC*-coordinating units to form a tetradentate *CNNC*- or *NCCN*-coordinating ligand has appealed to many researchers. Figure 1 summarizes just a selection of examples of Pt­(II) complexes of tetradentate ligands reported over the past 15 years, in which the two *NC*-ligating units are linked through a carbon atom,^10,11^ a nitrogen atom,^12^ or an oxygen atom.^13–15^ For a more thorough overview, the reader is directed to the recent comprehensive article on the subject by You.^9^ All of the complexes shown are luminescent in solution at room temperature, benefiting from the greater rigidity associated with the tetradentate ligand. Aside from tetradenticity, what these ligands have in common is that the central chelate ring (*i.e*., of the three chelate rings that form upon binding to the metal ion) is 6-membered, as opposed to 5-membered. The incorporation of a 6-membered chelate expands the binding site and allows the ligating atoms to attain roughly the same positions relative to the metal as those which they would occupy in the corresponding acyclic Pt­(*NC*)_2_ complex.[Fn fn1]

**Figure 1:**
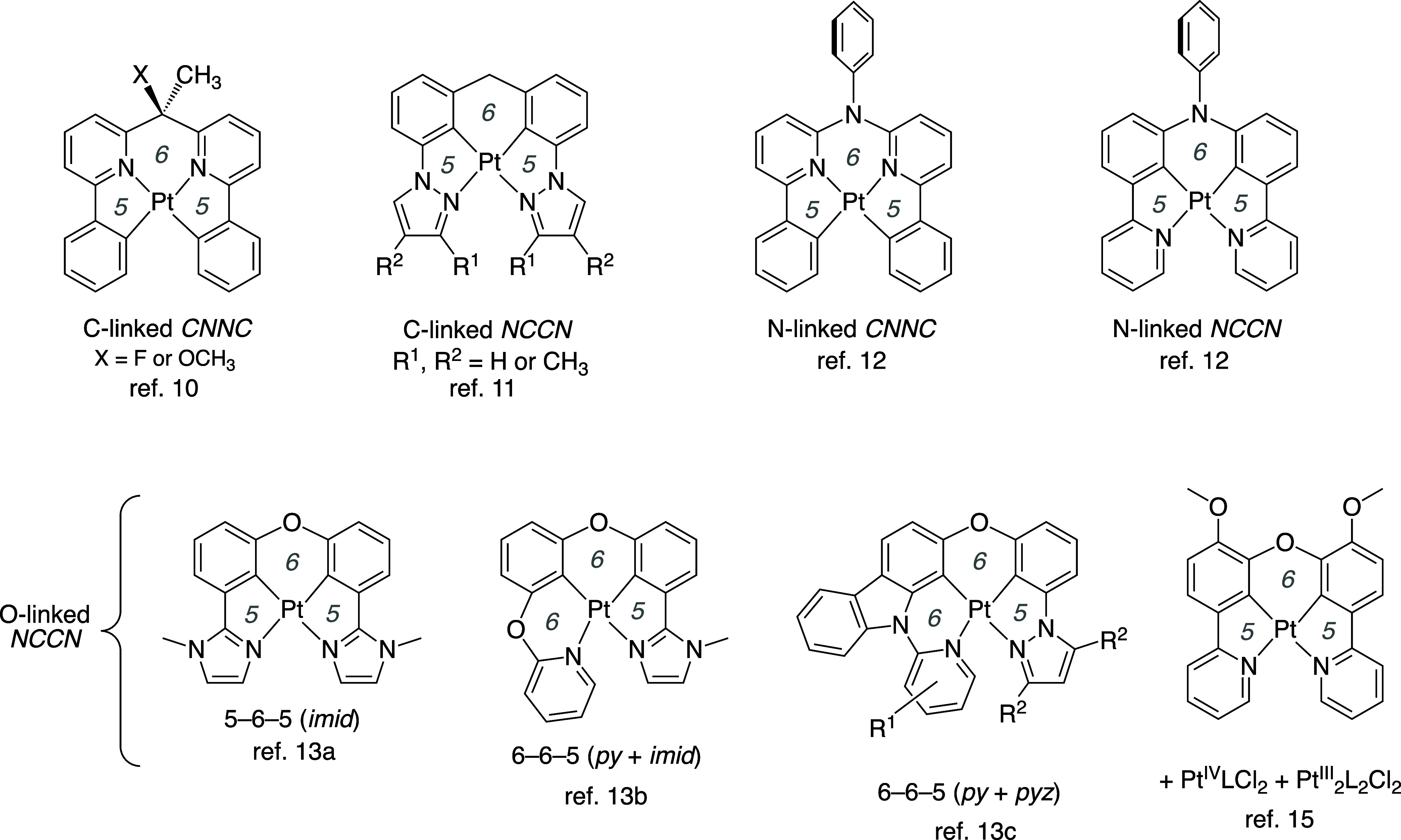
A representative selection of cyclometalated Pt­(II) complexes of tetradentate ligands that incorporate two NC-ligating units linked through a carbon, nitrogen, or oxygen atom. The italicized numbers in light font indicate the chelate ring size.

While it is the +2 oxidation state that has been the most explored in the context of luminescent complexes, the +4 state has been attracting increasing interest. Von Zelewsky already demonstrated in the 1980s the oxidation addition of alkyl halides RX to the Pt^II^(*NC*)_2_ complexes referred to above, to generate Pt^IV^(*NC*)_2_RX.^16^ Jenkins and Bernhard reported several compounds of the type [Pt­(*NC*)_2_(*NN*)]^2+^ in 2010, some of which were modestly luminescent.^17^ More recently, González-Herrero has shown how intense, long-lived, triplet emission may be obtained from a range of cyclometalated Pt­(IV) complexes.^18^ On the other hand, there are few instances of Pt­(III) complexes with cyclometallating ligands.^19^ Recent work by MacLachlan and co-workers on an oxygen-linked *NCCN* ligand is of particular interest in that respect: the Pt^II^L complex (Figure 1, bottom right) could be readily oxidized to Pt^IV^Cl_2_ in air in the presence of Cl^–^, but treatment of Pt^II^L with N-chloro-succinimide gave instead an unusual dinuclear Pt­(III) complex of the form Pt_2_L_2_Cl_2_, containing a Pt–Pt bond.^15,20^

In the present study, we sought to explore the cycloplatination of three new *NCCN*-coordinating ligand types based on 3,3′-bis­(2-pyridyl)­biphenyl (bpybpH_2_), illustrated schematically in Figure 2. We speculated that the poor bite angles and expected strain in the binding of bpybp to the metal in a tetradentate manner, generating three 5-membered chelate rings: structure type **A**, might render complexes of this ligand poorly emissive at best or even synthetically inaccessible altogether. Indeed, there appear to be no reported cyclometalated complexes featuring a 5–5–5 platinacycle. Thus, we chose to also examine two variants of **A**, in which either one or two of the pyridyl rings were replaced by 8-substituted quinolines: structure types **B** and **C**, respectively. The resulting complexes would feature two 5-membered and one 6-membered chelate, **B**, or one 5-membered and two 6-membered chelates, **C**, thus attenuating the strain and allowing a more optimal bite angle. Previous studies have shown that various *NNN*- and *NCN*-binding tridentate ligands containing an 8-substituted quinoline in place of a pyridine offer the Pt­(II) ion a more optimal bite, with angles between the *trans*-related ligating atoms closer to 180°.^21,22^ This in turn may lead to stronger ligand fields, enhanced rigidity in the excited state, and improved emission, an observation found also for other metal ions like Ru­(II)^23^ and Cr­(III).^24^ Note that previously reported examples of Pt­(II) complexes with tetradentate *NCCN* or *CNNC* ligands (*e.g*., those in Figure 1) feature a 6-membered chelate in the “central” position (5–6–5 or 5–6–6 platinacycles), whereas the 6-membered chelate(s) are in the lateral position in **B** and **C** (*i.e*., 6–5–5 and 6–5–6, respectively).

**Figure 2:**
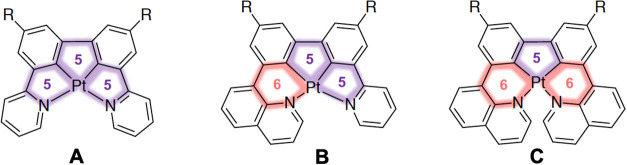
Three classes of Pt­(II) complexes investigated in this work: **A** formed from the ligand 3,3′-bis­(2-pyridyl)­biphenyl or its t-butyl derivative (*R* = H or *t*-Bu) and featuring a 5–5–5 platinacycle; and **B** and **C** analogues in which one or two of the pyridines are replaced by an 8-substituted quinoline, to give 6–5–5 and 6–5–6 platinacycles, respectively (*R* = *t*-Bu).

## 2 Results and Discussion

### 2.1 Synthesis

The parent proligand 3,3′-di­(2-pyridyl)­biphenyl H_2_L^0^ was originally prepared by Geest and Steel through the nickel-catalyzed homocoupling of 2-(3-bromophenyl)­pyridine, **ppy-Br**. We started from the same precursor but chose instead to convert a portion of it to the pinacol boronate ester **ppy-B** by palladium-catalyzed reaction with bis­(pinacolato) diboron (as this approach would then also offer a route to nonsymmetrical analogues such as H_2_L^2^). The subsequent palladium-catalyzed Suzuki cross-coupling of **ppy-Br** with **ppy-B** led cleanly to H_2_L^0^ in very good yield (Scheme 1). The same approach was applied to the *tert*-butyl derivative[Fn fn2] of the precursor (ppy*-Br, prepared by Stille coupling, as described in the Supporting Information), giving H_2_L^1^ in yet higher yield. The corresponding, symmetrical, quinoline-based proligand H_2_L^3^ was prepared by the same strategy from 8-(3-bromo-5-*t*-butyl-phenyl)­quinoline pqu*-Br (prepared from quinoline-8-boronic acid as illustrated in Scheme 1 and described in the Supporting Information). Finally, the nonsymmetrical proligand H_2_L^2^, incorporating one pyridine and one quinoline, was obtained by the cross-coupling of pqu*-Br and ppy*-B.

**Scheme 1:**
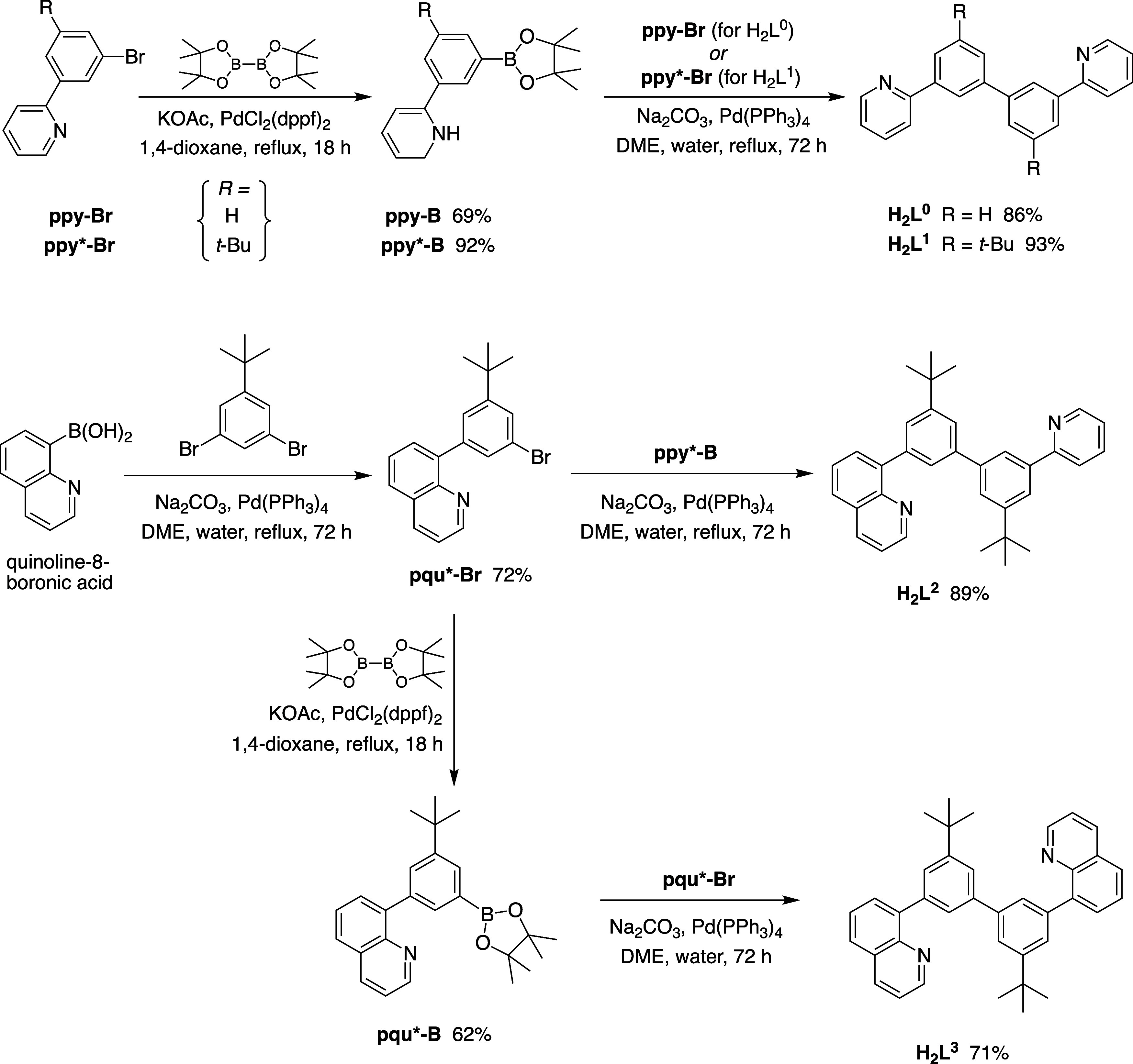
Synthetic Route to Ligands H_2_L^0^, H_2_L^1^, H_2_L^2^ and H_2_L^3^

### 2.2 Platination Reactions

The cyclometalation of arylpyridine ligands with platinum­(II) is typically achieved by reaction with K_2_PtCl_4_ in acetic acid at reflux. For H_2_L^0^, these conditions led to a yellow solid, which had such poor solubility that it could not be characterized. We did, however, unexpectedly isolate a single crystal of the Pt­(IV) complex PtL^0^Cl_2_, identified by crystallography (Section 4.3). Owing to the poor solubility, we turned our attention to *tert*-butyl derivatives. The quinoline-containing proligands H_2_L^2^ and H_2_L^3^ gave mixtures of Pt­(II) and Pt­(IV) complexes, PtL^2–3^ and PtL^2–3^Cl_2_, respectively, as identified by ^1^H NMR spectroscopy. Attempts to isolate the pure Pt­(II) complexes from these mixtures were thwarted by a tendency for them to oxidize readily in solution. On the other hand, the Pt­(IV) complexes were successfully isolated as yellow solids, either using column chromatography in the case of PtL^2^Cl_2_ or by recrystallization from CH_2_Cl_2_/hexane for PtL^3^Cl_2_ (Scheme 2). The formation of Pt­(IV) complexes, despite the use of deoxygenated conditions, is not unprecedented. For example, Ortiz et al. noted the formation of some Pt^IV^(*NCN*-dphenb)­Cl_3_ during the synthesis of Pt^II^(dphenb)Cl from K_2_PtCl_4_ {dphenb = 1,3-di­(2-trifluoro-methyl-4-phenanthridinyl)­benzene};^21b^ Bruce and co-workers observed the spontaneous formation of *cis*-Pt­(*NC*)­Cl_2_ compounds using the same Pt­(II) salt;^25^ and Zhang et al. isolated *trans*-Pt­(*NCCN*)­Cl_2_ complexes,^26^ a type subsequently exploited as precursors to Pt­(II) complexes for OLEDs by Allison et al.^27^ In 2020, Soto et al. obtained a mixture of Pt­(II), Pt­(III) and Pt­(IV) compounds with the *NCCN* ligand denoted “ref 15.” in Figure 1.^15^ Adventitious oxygen was responsible. In the present instance, we noted that the oxidation appeared possible also during the workup and purification.

**Scheme 2:**
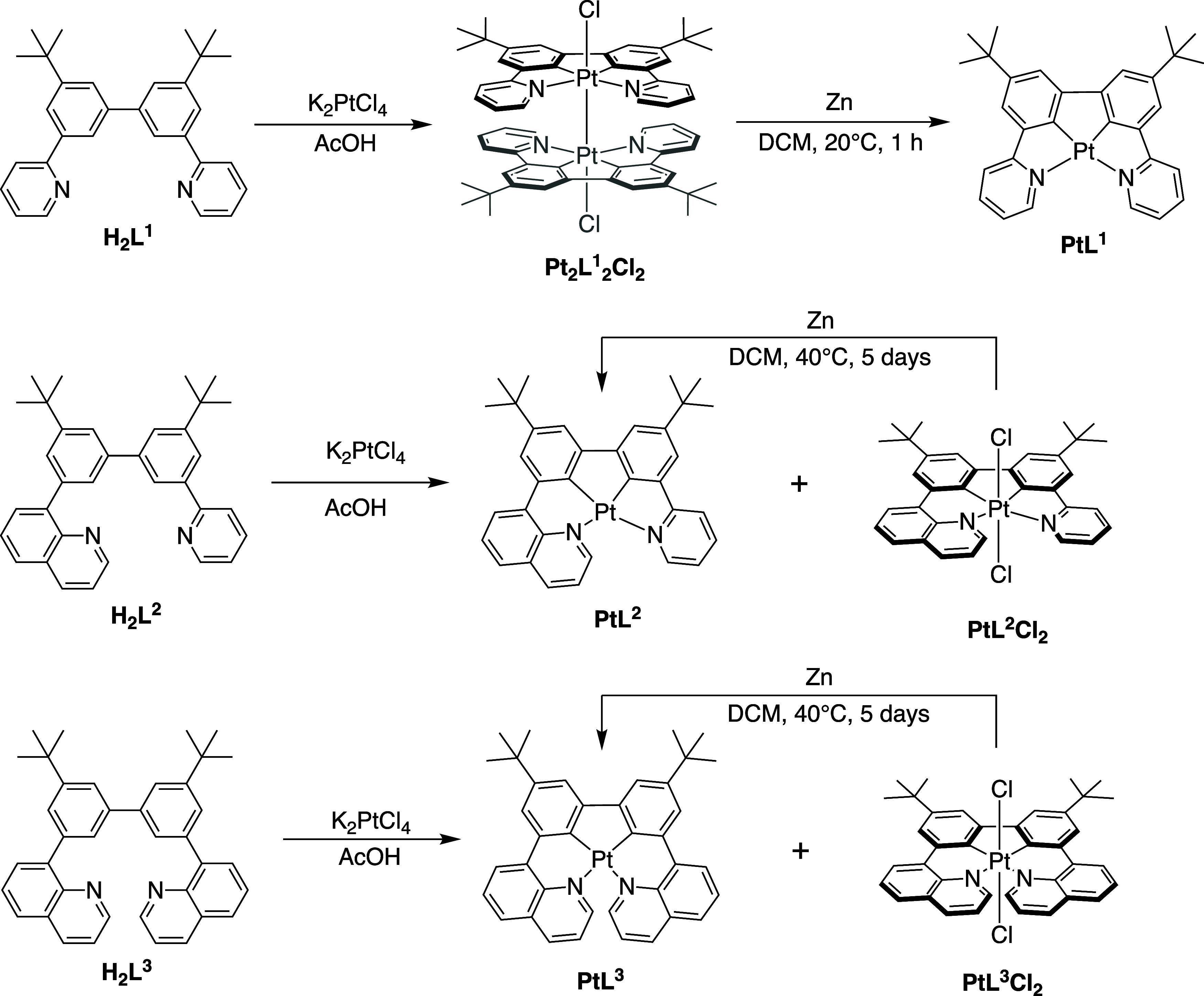
Illustrating the Outcome of the Reactions of the Proligands H_2_L^1–3^ with K_2_PtCl_4_ ^a^

a For H_2_L^1^, the dinuclear Pt­(III) complex Pt_2_L^1^_2_Cl_2_ was formed, from which the Pt­(II) complex PtL^1^Cl was subsequently obtained by reduction with Zn. For H_2_L^2^ and H_2_L^3^, mixtures of the Pt­(II) and Pt­(IV) complexes were obtained; subsequent reduction of the latter with Zn gave the former.

We found that pure samples of the Pt­(II) complexes could be obtained indirectly, by reduction of the Pt­(IV) complexes with activated zinc in dichloromethane solution heated at reflux over a period of 5 days, following a similar approach to that used by Soto et al.^15^ The reduction of the Pt­(IV) compounds to their Pt­(II) analogues is accompanied by a modest upfield shift of the ^1^H NMR resonances, in line with the more shielded nature of the protons in the lower oxidation state complex (Figure 3).

**Figure 3:**
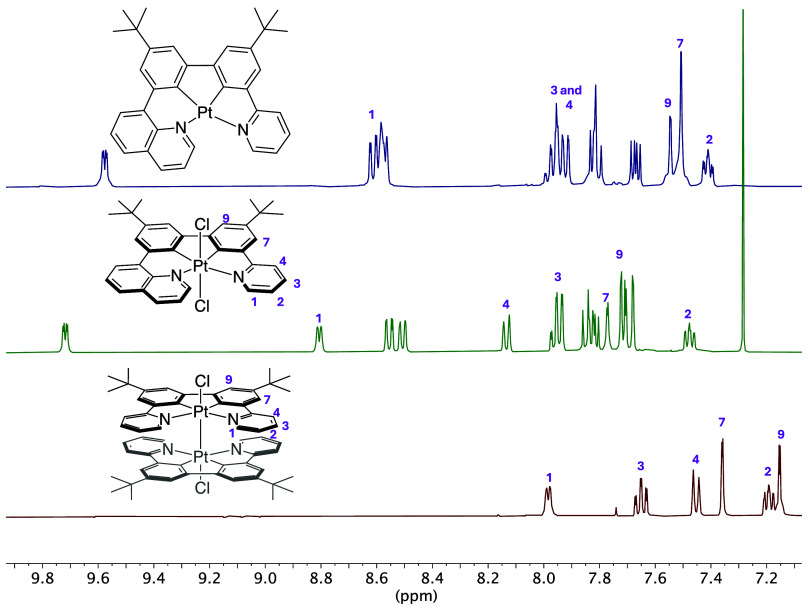
^1^H NMR spectra of complexes PtL^**2**^Cl, PtL^**2**^Cl_2_, and Pt_2_L^1^_2_Cl_2_ in CD_2_Cl_2_ at 400 MHz.

The reaction of the pyridine-based proligand H_2_L^1^ with K_2_PtCl_4_ in acetic acid gave a result different from that observed with the quinoline-containing compounds H_2_L^2^ and H_2_L^3^. In this case, the ^1^H NMR spectrum of the product shows a set of resonances that are shifted significantly upfield relative to those of PtL^2^ (let alone PtL^2^Cl_2_). Considering the ppy unit that is common to both L^**1**^ and L^**2**^, similar shifts would be anticipated for PtL^1^ to those for the ppy resonances of PtL^2^; the upfield shift is thus not consistent with the product being PtL^1^. The product was subsequently identified by X-ray crystallography (*vide infra*) as a dinuclear Pt­(III) complex, Pt_2_L^1^_2_Cl_2_, reminiscent of that reported by Soto et al., referred to in the introduction.^15^ The anomalous upfield shift in ^1^H NMR evidently results from the ring current effect of π–π stacking between the ligands. The fact that the product for H_2_L^1^ stops at the +3 oxidation state, whereas H_2_L^2^ and H_2_L^3^ led to a significant proportion of the Pt­(IV) complex, may be due to the closer approach of the Pt centers that is possible for the L^1^ ligand, to form the Pt–Pt bond, and to the intramolecular π stacking further enhancing the stability of the dinuclear compound. The Pt­(*NCCN*) units for this ligand are essentially planar, whereas those for L^2^ and L^3^ are puckered, as they feature 6-membered chelates (see Figures 4 and 5 and discussion of X-ray crystallography below). There is no evidence of the rotation of the Pt­(*NCCN*) planes relative to one another in solution. The stability of Pt_2_L_2_^1^Cl_2_ to oxidation is further manifest in that treatment with the oxidizing/chlorinating agent PhICl_2_ — a reagent widely used to obtain Pt­(IV) complexes from Pt­(II)—failed to give any PtL^1^Cl_2_. On the other hand, the treatment of the Pt­(III) complex Pt_2_L^1^_2_Cl_2_ with activated zinc at room temperature led to rapid reduction to the Pt­(II) complex, PtL^1^, which was subsequently isolated and fully characterized.

**Figure 4:**
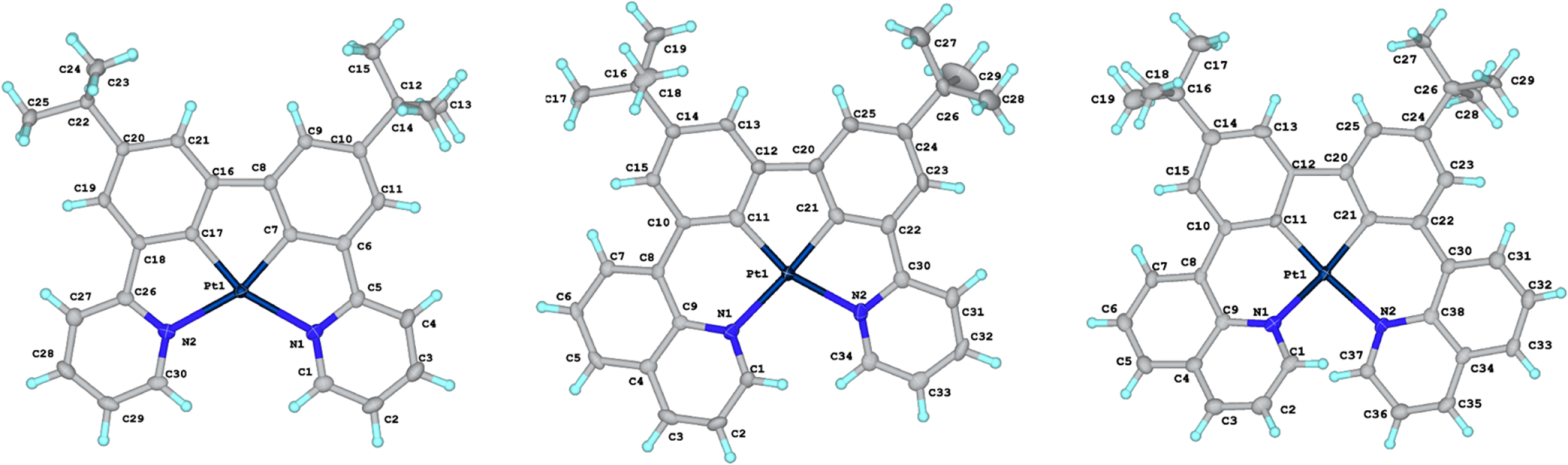
Molecular structures of the Pt­(II) complexes PtL^1^, PtL^2^, and PtL^3^.

**Figure 5:**
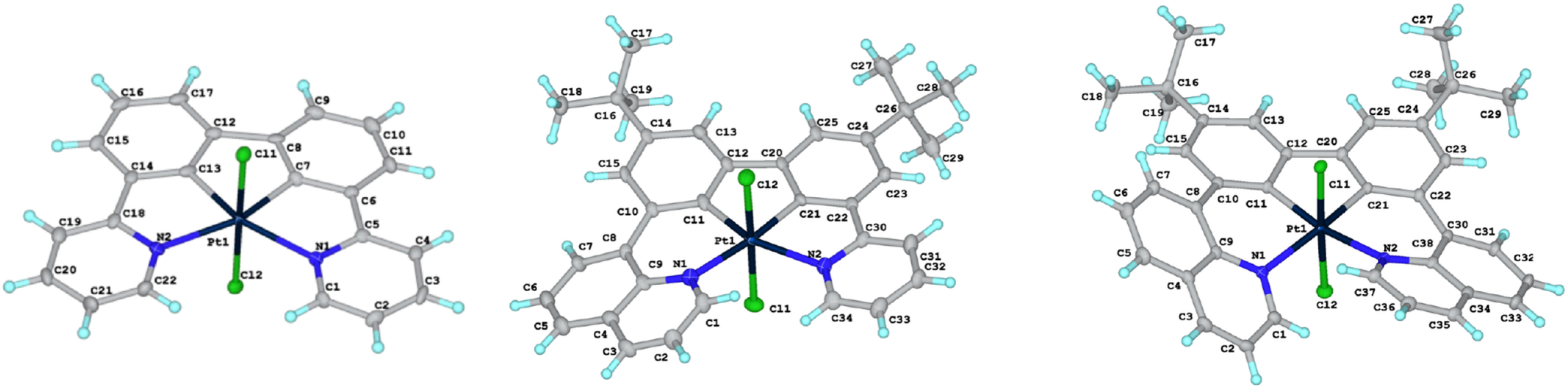
Molecular structures of the Pt­(IV) complexes PtL^0^Cl_2_, PtL^2^Cl_2_, and PtL^3^Cl_2_.

### 2.3 Structural Characterization in the Solid State

X-ray diffraction analysis was carried out on single crystals of all of the Pt­(II), Pt­(III), and Pt­(IV) complexes. The molecular structures are shown in Figures 4–6, and selected bond lengths and angles are summarized in Table 1. The d^8^ Pt­(II) complexes show the expected pseudosquare planar geometries around the Pt­(II) ion, while the d^7^ Pt­(III) and d^6^ Pt­(IV) complexes show pseudo-octahedral geometries. For the complexes containing pyridyl rings, the angle between the pyridine N and the metalated carbon *trans* (*i.e*., spanning two 5-membered chelate rings) is around 160°. Such a deviation of *trans*-related ligating units from linearity is very typical of ligands like *NNN*-terpyridine, *NNC*-2-phenylpyridine, *NCN*-dipyridylbenzene, and *NCC*-pyridylbiphenyl that form two 5-membered chelate rings when they bind to the metal ion. The deviation from linearity is smaller for related 8-quinolyl-based ligands that bind through 6-membered chelates, such as *NNN*-bis­(8-quinolyl)­pyridine and *NCN*-bis­(8-quinolyl)­benzene.^21^ Indeed, in the present instance, the corresponding angles between the quinolyl N and the *trans*-disposed carbon are in the range 169–174°. The attainment of such an angle does require a rotation of the plane of the quinolyl ring relative to the neighboring benzene ring, since the metal ion would be too large to bind in a planar conformation. Interplanar angles are listed in Table 1. The quinolyl-based complexes are thus chiral through helical wrapping of the ligand around the metal ion. PtL^2^, PtL^3^Cl_2_, and PtL^3^ all crystallize in the centrosymmetric space group P-1, and the crystals accordingly comprise a racemic mixture of the two enantiomers. In contrast, PtL^2^Cl_2_ crystallizes in the noncentrosymmetric *P*2_1_2_1_2_1_ space group, so the crystal comprises a single enantiomer.

**Figure 6:**
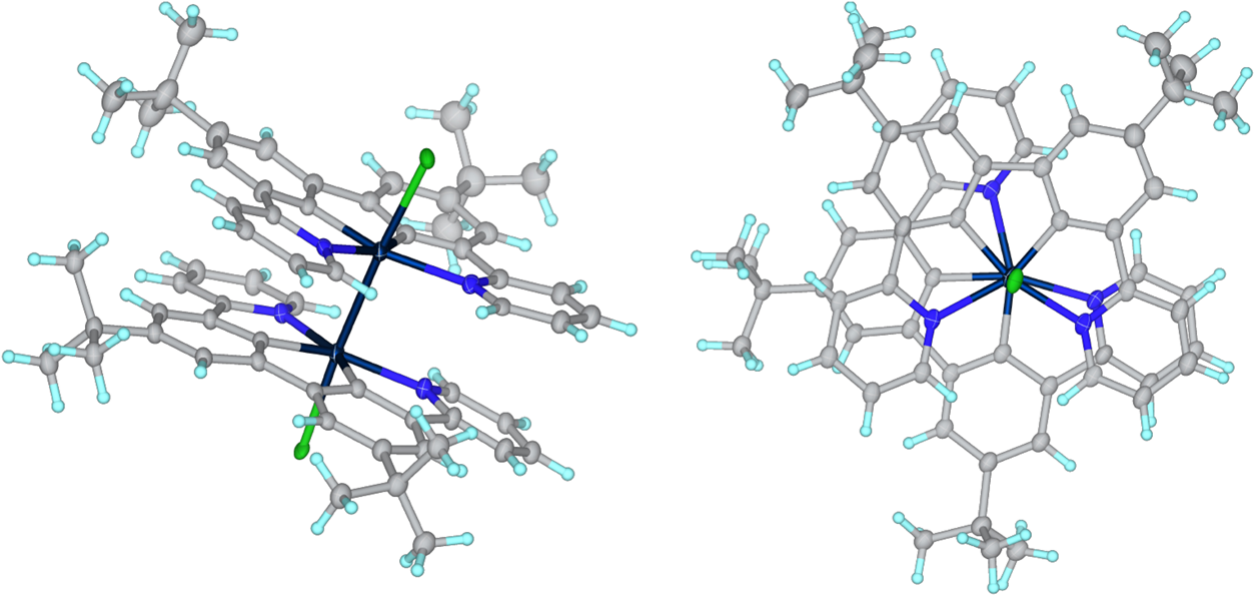
Two views of the molecular structure of dinuclear Pt­(III) complex Pt_2_L^1^_2_Cl_2_: side-on view (left) and looking down the Pt–Pt bond (right).

**Table 1:** Selected Bond Lengths (Å), and Bond and Interplane Angles (°), for the Pt­(II) Complexes PtL^1–3^, the Pt­(III) Complex Pt_2_L^1^_2_Cl_2_, and the Pt­(IV) Complexes PtL^2–3^Cl_2_ ^a^

	**PtL** ^**1**^	**PtL** ^**2**,^ ^b^	**PtL** ^**3**^	**Pt**_**2**_ **L^1^**_**2**_ **Cl**_**2**_	**PtL** ^**0**^ **Cl**_**2**_	**PtL** ^**2**^ **Cl**_**2**_	**PtL** ^**3**^ **Cl**_**2**_
CCDC no.	2484707	2484711	2484706	2484708	2484709	2484712	2484710
Pt–C^trans to py^	1.935(3)/1.927(3)	1.964(7)/1.967(7)		1.955(16)/1.950(18)/1.997(15)/1.910(17)	1.952(5)/1.969(5)	1.990(8)	
Pt–N^py^	2.177(2)/2.178(2)	2.161(6)/2.162(5)		2.220(13)/2.184(13)/2.210(15)/2.156(12)	2.201(4)/2.203(4)	2.282(7)	
Pt–N^quin^		2.105(6)/2.086(5)	2.082(9)/2.143(8)			2.166(7)	2.1786(15)/2.1693(14)
Pt–C^trans to quin^		1.942(7)/1.922(6)	1.967(10)/1.961(10)			1.964(7)	1.9936(16)/1.9896(17)
Pt–Cl				2.453(3)/2.481(3)	2.3114(13)/2.3191(13)	2.3089(19)/2.3176(19)	2.3071(4)/2.3285(4)
Pt–Pt				2.6686(7)			
C^trans to py^–Pt–N^py^	159.9(1)/159.7(1)	161.2(2)/160.4(2)		161.7(7)/159.9(6)/159.3(6)/161.4(6)		159.9(3)	
N^quin^–Pt–C^trans to quin^		173.4(3)/167.6(3)	171.4(4)/170.8(4)			173.8(3)	
	170.25(6)/169.16(6)						
Cl–Pt–Cl					178.45(5)	177.05(8)	177.195(18)
Cl–Pt–Pt				175.83(10)/176.10(9)			
interplane ph ∧ ph	4.54(8)	5.3(3)/8.4(2)	6.6(3)	10.4(6)/9.5(6)	6.19(17)	5.5(3)	8.85(5)
interplane ph ∧ py	2.13(8)/5.17(9)	5.8(3)/14.0(3)		5.7(6)/11.8(6)/3.0(5)/10.2(6)	5.29(18)/1.72(17)	8.9(3)	
interplane ph ∧ quin		23.4(2)/26.75(19)	26.2(3)/25.8(3)			18.9(2)	23.59(4)/23.31(4)

a The planes defined by the phenyl, pyridine, and quinoline units of the ligand are abbreviated as ph, py, and quin, respectively.

b The two different molecules in this structure are quite different, the helical twist being more pronounced in the second, possibly due to differences in intermolecular π stacking between the quinoline rings of neighboring molecules.

The structures of these new complexes, featuring 5–5–5, 6–5–5, and 6–5–6 platinacycles, may be compared with 5–6–5 and 6–6–5 examples from Figure 1 (where structures are available). In the crystallographically characterized examples of the former, the deviations of the metal coordination plane from planarity are generally small; that is, when the 6-membered ring is central, the Pt­(*NC*) planes can occupy roughly the same plane. A degree of steric interaction between the rings occupying the “open ends” does lead to a small twisting of the two Pt­(*NC*) planes relative to one another, although one of the three independent molecules of the unit cell of the Pt­(*NCCN*) system reported by Huo and colleagues (Figure 1) is essentially flat.^12^ On the other hand, the presence of two mutually adjacent 6-membered chelates leads to substantial puckering; for example, in the structurally characterized pyrazolyl analogue of the complex labeled ref 13b in Figure 1, there is a large distortion from planarity in the lateral (Pt–N–C–O–C–C) ring.^13b^ The Pt–C bond lengths in the new Pt­(II) complexes are slightly shorter than those of *cis*-Pt­(ppy)_2_ {mean = 1.988(25) Å}^6a^ while the Pt–N^py^ bonds are slightly longer than in *cis*-Pt­(ppy)_2_ {mean = 2.127(4) Å},^6a^ consistent with the *CC*-chelating unit being incorporated into the “interior” of the *NCCN* chelate. The Pt–N^quin^ bonds are marginally shorter than the Pt–N^py^.

The Pt–N and Pt–C bonds are marginally, but consistently, longer in the Pt­(IV) compounds than in the corresponding Pt­(II) compounds. This trend is in line with our previous structural studies of, for example, Pt­(*NNC*)­Cl_3_ versus Pt­(*NNC*)Cl (where *NNC* represents 2-phenylpyridine or a derivative thereof), where we speculated that it might be due to a slight expansion being required to accommodate the additional ligands at the “axial” positions.^28^ The Pt–Pt bond in Pt_2_L^1^_2_Cl_2_ is unequivocal at 2.6686(8) Å, compared to 3.5 Å for the sum of the van der Waals radii for two Pt atoms. The nearest distance between the planes defined by the two PtL^1^ units is 3.306(5) Å, indicating significant π–π interactions, as also concluded above from the upfield-shifted ^1^H NMR resonances.

### 2.4 Absorption Spectra of the Complexes

The absorption spectra of the complexes are shown in Figure 7, and the corresponding numerical data are collated in Table 2. Considering first the Pt­(II) complexes (Figure 7a), their spectra are quite typical of cyclometalated complexes based on arylpyridines. Intense transitions at wavelengths <300 nm, due to intraligand π–π* transitions, are accompanied by somewhat less intense bands in the visible region that have no counterparts in the free ligands. The latter are typically assigned to transitions of d_Pt_ | π_NC_ → π*_NC_ character (^1^MLCT/^1^LLCT), and such an assignment is supported here too by DFT calculations (see Section 2.5 below). It is notable, however, that the λ_max_ of 458 nm for the bis-pyridine complex PtL^1^ is substantially red-shifted compared not only to the corresponding bis-bidentate complex *cis*-Pt­(ppy)_2_ (λ_max_ = 402 nm in the same solvent), but also to the complexes of Figure 1 wherein the *NC*-chelating units are linked through an intervening atom (C, N, or O). Evidently, the enhanced conjugation associated with the biphenyl unit of L^1^ (*i.e*., interannular connection of the two *NC* units as opposed to through a spacer atom) lowers the energy of the pertinent excited state. For further comparison, the lowest-energy band of Pt­(bph)­(py)_2_—featuring a *CC*-coordinated biphenyl (bph) but separate monodentate pyridines—is a shoulder at 375 nm.^29^ The replacement of one of the pyridines by the quinoline in PtL^2^ leads to a further red-shift of the lowest-energy band to 484 nm, and then to 501 nm in the bis-quinoline complex PtL^3^ (a reduction in energy of 1200 and 700 cm^–1^, respectively), reflecting the more extended conjugation associated with the quinolines.

**Figure 7:**
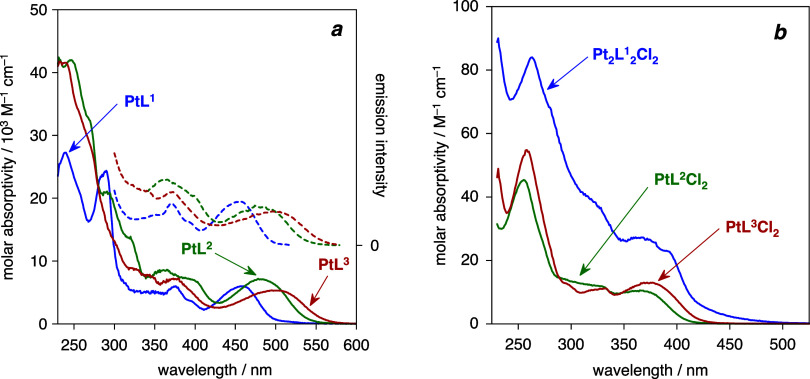
UV–vis absorption spectra of (a) the platinum­(II) complexes PtL^**1**–**3**^ (blue, green, and red solid lines, respectively) with the photoluminescence excitation spectra also shown as the correspondingly colored dashed lines (λ_em_ = 570 nm for PtL^1^ and 680 nm for PtL^2–3^Cl) and (b) the platinum­(III) complex Pt_2_L^1^_2_Cl_2_ (blue) overlaid with the platinum­(IV) complexes PtL^**2**^Cl_2_ and PtL^**3**^Cl_2_ (red and green, respectively). The spectra shown were recorded at concentrations of approximately 10^–5^ M in a CH_2_Cl_2_ solution at 295 K.

**Table 2:** Photophysical Properties of the Pt­(II) Complexes PtL^1–3^, the Pt­(III) Dimer Pt_2_L^1^_2_Cl_2_, and the Pt­(IV) Complexes PtL^2–3^Cl_2_

	**absorption at 295 K** ^a^	**emission at 295 K** ^a^,^b^					**emission at 77 K** ^c^	
**complex**	**λ**_**max**_/nm (ε/M^**–1**^ **cm** ^**–1**^ **)**	**λ**_**max**_ **/nm**	**Φ**_**lum**_ **/%** ^d^	**τ/μs** ^e^	***k***_**r**_ **/10** ^**3**^ **s** ^**–1**,^ ^f^	**∑** ***k***_**nr**_ **/**10^**3**^ **s** ^**–1**,^ ^f^	**λ**_**max**_ **/nm**	**τ/μs** ^g^
**PtL** ^**1**^	239 (27,300), 284sh (23,400), 290 (24,400), 357 (5700), 385sh (3640), 458 (6000)	534, 570	67	3.5	190	93	523, 563, 605	11
**PtL** ^**2**^	231 (24,400), 248 (41,900), 269sh (32,500), 291 (21,100), 322 (13,200), 361 (9000), 401 (6750), 484 (7120)	670	2.2	2.5	8.6	390	613, 668	12
**PtL** ^**3**^	233 (41,700), 241 (41,500), 324 (8590), 345 (7490), 376 (7150), 501 (5310)	700	0.4	0.65	5.7	1530	631, 690	12
**Pt**_**2**_ **L^1^**_**2**_ **Cl**_**2**_	231 (90,000), 263 (84,000), 324sh (37,600), 371 (26,500), 393sh (22,700)	^h^					^i^	
**PtL** ^**2**^ **Cl**_**2**_	255 (45,300), 297 (13,900), 329 (11,900), 366 (10,500)	^h^					525, 551, 564sh, 600, 655	270
**PtL** ^**3**^ **Cl**_**2**_	258 (54,800), 297sh (12,500), 331 (11,100), 375 (13,000)	^h^					521, 565, 616	300

a In CH_2_Cl_2_.

b In a degassed solution.

c In diethyl ether/isopentane/ethanol (2:2:1 v/v).

d Photoluminescence quantum yield measured using [Ru­(bpy)_3_]­Cl_2_ in water as the standard, for which Φ_lum_ = 4.0%.

e Lifetimes at 295 K were measured by multichannel scaling following excitation at 404 nm with a pulsed-diode laser.

f Radiative *k*_r_ and nonradiative ∑*k*_nr_ rate constants estimated assuming that the emitting state is formed with unit efficiency such that *k*_r_ = Φ_lum_/τ and ∑*k*_nr_ = (1−Φ_lum_)/τ.

g Lifetimes at 77 K were measured by multichannel scaling following excitation with a μs-pulsed xenon lamp at 400 nm.

h No detectable emission at 295 K.

i No detectable emission at 77 K.

The spectra of the Pt­(IV) complexes contrast with those of their Pt­(II) analogues in that the lowest-energy absorption bands are greatly blue-shifted (λ_max_ = 366 and 375 nm for PtL^2^Cl_2_ and PtL^3^Cl_2_, respectively; Figure 7b). This trend is consistent with previous observations of cyclometalated Pt­(IV) complexes when compared to their Pt­(II) analogues.^16–19,28^ It can be readily interpreted through the decrease in the energy of the d orbitals with increasing oxidation state, which in turn lowers the energy of the metal-centered orbitals in the ^1^[d_Pt_ | π_Ar_ → π*_NN_] transitions, thus increasing the absorption energy. The slightly lower energy of the absorption band of PtL^3^Cl_2_ versus PtL^2^Cl_2_ (≈ 700 cm^–1^) accompanying the introduction of the second quinoline unit mirrors the trend for the Pt­(II) complexes. The spectrum of the dinuclear Pt­(III) complex is similar in profile to that of the Pt­(IV) complexes but exhibits higher molar absorptivities (roughly double) across the entire wavelength range (Figure 7b). This can obviously be attributed to the presence of two conjugated Pt­(*NCCN*) units per molecule of Pt_2_L^1^_2_Cl_2_.

### 2.5 Computational Study

Density functional theory (DFT) and time-dependent density functional theory (TD-DFT) calculations with the Tamm-Dancoff approximation (TDA) were performed on the complexes. Geometry optimizations in the ground state were performed using B3LYP/def2-TZVP. Details are provided in the Supporting Information. As anticipated, the HOMO is located on the phenyl rings of the ligand and on the Pt center in each case, while the LUMO is on the pyridine ring for complex PtL^1^ and on the quinoline ring(s) for complexes PtL^2–3^ {Figure 8, and corresponding T_1_ spin density plot in Figure S25}. The TD-DFT calculations show that the lowest-energy, spin-allowed transitions for the three Pt­(II) complexes are predominantly HOMO → LUMO in character (>95%), with oscillator strengths *f* > 0.16.

**Figure 8:**
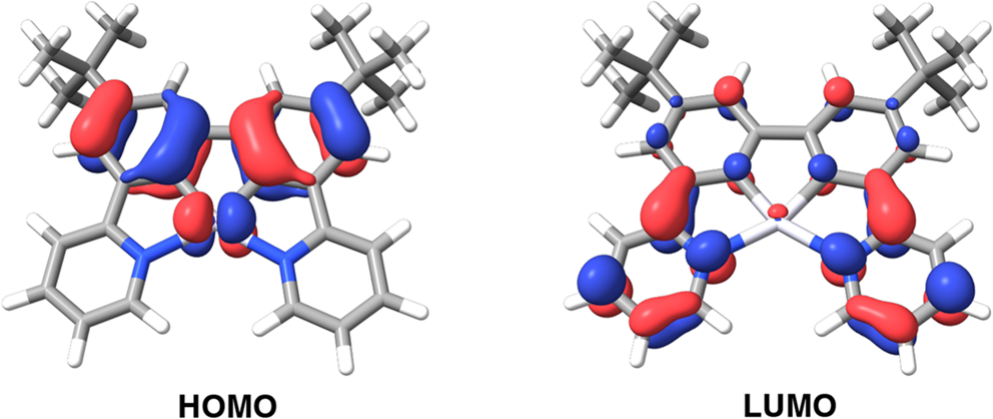
Frontier orbital plots for PtL^1^ at the S_0_ geometry.

The frontier orbital energy level diagrams for PtL^2^, PtL^3^, PtL^2^Cl_2_, and PtL^3^Cl_2_ are shown in Figures 9 and S26–S29, confirming that the HOMOs are again primarily based on the biphenyl units in the Pt­(IV) as well as the Pt­(II) complexes, with the LUMOs on the quinoline(s). For PtL^2^Cl_2_, the lowest singlet transition primarily involves contributions from the HOMO and the LUMO+1 orbitals (78%), with the latter lying along the Pt–Cl bonds. Similarly, the corresponding transition for PtL^3^Cl_2_ is characterized by contributions from the HOMO → LUMO (40%) and HOMO → LUMO+2 (50%), with the LUMO+2 based along the Pt–Cl bonds. The HOMO of each Pt­(IV) complex is approximately 0.7 eV lower than that of its Pt­(II) analogue, reflecting the increase in oxidation state and the lowering of the metal d orbital energies alluded to above. The larger energy gaps in the Pt­(IV) complexes are consistent with the experimentally observed blue shifts.

**Figure 9:**
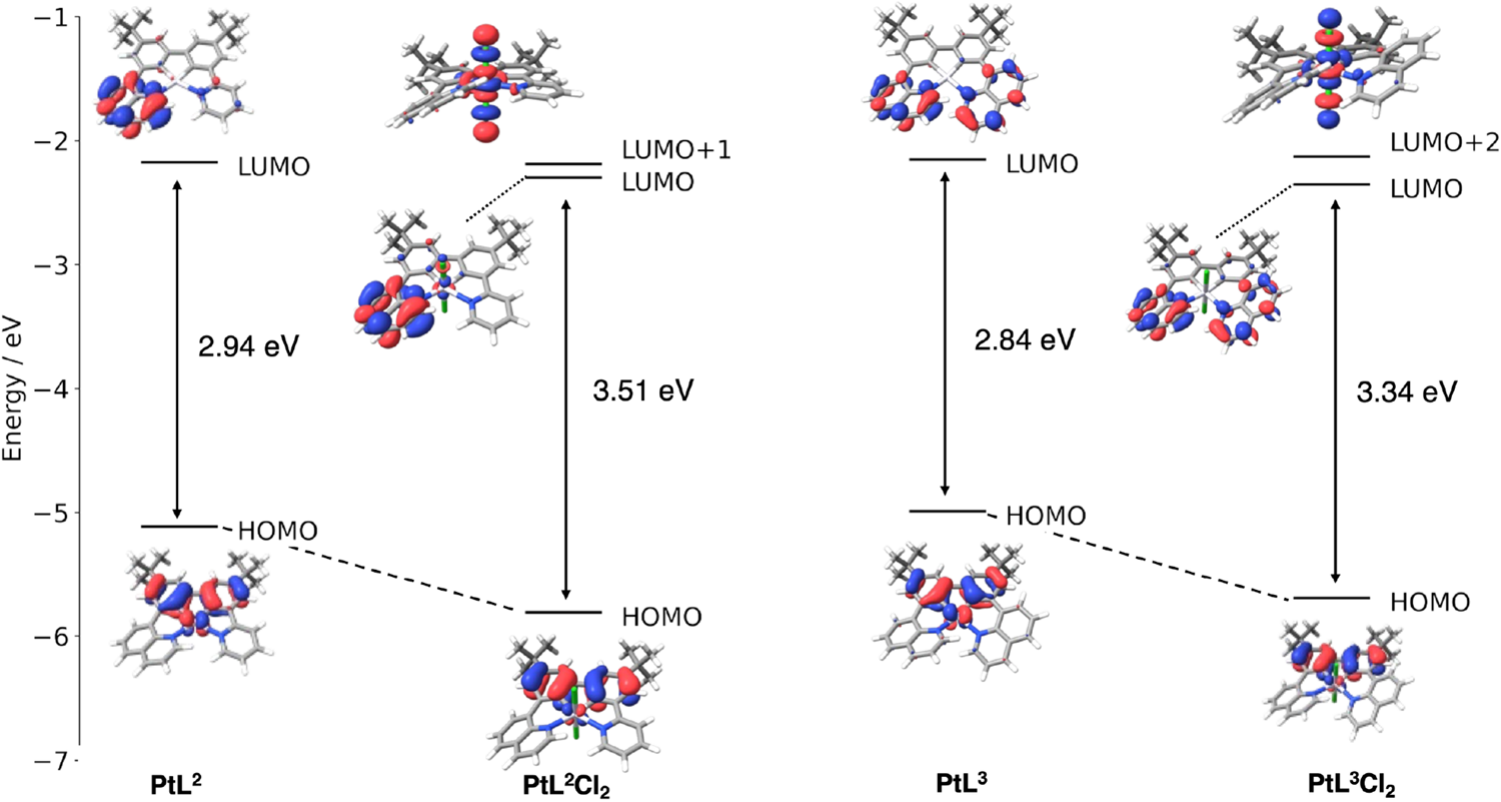
Molecular orbital diagrams showing pertinent frontier orbitals of PtL^2^, PtL^3^, PtL^2^Cl_2_, and PtL^3^Cl_2_, together with their energies.

### 2.6 Photoluminescence Studies

The Pt­(II) complexes are all photoluminescent in a deoxygenated dichloromethane solution at room temperature (Figure 10), and the excitation spectra closely match the absorption spectra (Figure 7a). Under these conditions, PtL^1^ emits in the green region of the spectrum and shows some vibrational structure, whereas the quinoline complexes PtL^2–3^ emit in the deep-red, and their spectra are broader and unstructured. PtL^3^ is a little red-shifted compared to PtL^2^ by around 600 cm^–1^, as in absorption. The spectra in a frozen glass at 77 K are all blue-shifted compared to room temperature, and the vibrational structure now becomes resolved for the quinoline complexes, too. The lifetimes at 77 K are around 10 μs, which is typical of formally forbidden phosphorescence in cyclometalated Pt­(II) complexes being promoted by the spin–orbit coupling (SOC) associated with the heavy metal.

**Figure 10:**
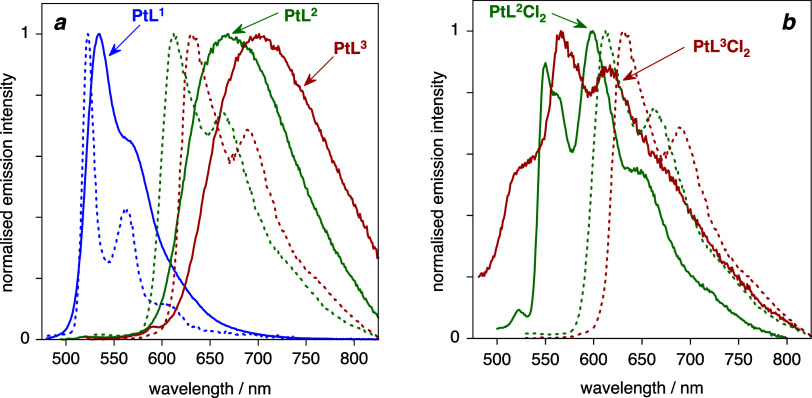
Emission spectra of (a) the Pt­(II) complexes PtL^1–3^ in CH_2_Cl_2_ at 295 K (blue, green, and red solid lines, respectively) and at 77 K in EPA (correspondingly colored dashed lines) and (b) the Pt­(IV) complexes PtL^2^Cl_2_ and PtL^3^Cl_2_ at 77 K in EPA (green and red solid lines respectively, with the spectra of the corresponding Pt­(II) complexes shown again as dotted lines to aid comparison; EPA = diethyl ether/isopentane/ethanol, 2:2:1 v/v).

In solution at 295 K, there is more variation in the lifetime and even more so in the luminescence quantum yields, Φ_lum_. PtL^1^ emits brightly, with Φ_lum_ = 67%, compared to 2.5 and 0.65% for PtL^2^ and PtL^3^. The trend of weaker emission for deep-red compared to green-emitting complexes is commonly encountered, reflecting the more efficient nonradiative decay expected for low-energy emitters through energy transfer into vibrations (the so-called “energy gap law”). The rate constants of nonradiative ∑*k*_r_ and radiative decay *k*_r_ estimated from the lifetimes and quantum yields (Table 1) reveal that the emissive states of PtL^2–3^ are indeed subject to faster nonradiative decay than PtL^1^. A plot of ln ∑*k*_nr_ against emission energy (based on λ_max_) shows a good correlation, notwithstanding the limited number of compounds and discontinuity expected upon the introduction of a quinoline in place of a pyridine (Figure S30). However, it is also clear that the *k*_r_ values are more than an order of magnitude lower for the red-emitting, quinoline-containing complexes than for PtL^1^. Such a trend has been noted previously with increasingly conjugated ligands: as the conjugation in the ligand becomes more extended, the filled ligand orbitals rise in energy, potentially resulting in less efficient mixing with the metal orbitals, and hence less efficient SOC and a lower *k*_r_ for triplet emission.^30^ In the present instance, such an effect is probably compounded by the out-of-plane distortion and the longer Pt–C bonds of the quinoline-containing complexes compared to PtL^1^.

The Pt­(IV) complexes do not exhibit detectable emission in deoxygenated solution at 295 K. At 77 K, however, PtL^2^Cl_2_ does give a clear-cut, vibrationally resolved spectrum, λ_0,0_ = 525 nm, and PtL^3^Cl_2_ emits weakly, too (Figure 10b). The emission in both cases is strongly blue-shifted compared to that of the Pt­(II) analogues under the same conditions (Figure 10b), in line with the absorption spectra and computational results, and interpreted in terms of the lowering in energy of the metal orbitals. Moreover, the luminescence lifetimes of the Pt­(IV) complexes are over an order of magnitude longer than those of the Pt­(II) analogues at around 300 μs (Table 2). This observation is again consistent with poorer mixing of the lower-energy d orbitals with the ligand orbitals, reducing the efficacy of SOC and suppressing the *k*_r_ values. Population analyses of the pertinent excited states confirm that the metallic contribution is much smaller in the Pt­(IV) complexes (Table S4). Moreover, as noted in Section 2.5, the Pt­(IV) complexes feature low-lying excited states that involve the population of antibonding orbitals oriented along the Pt–Cl bonds. Such LMCT states have previously been implicated in promoting nonradiative decay in Pt­(IV) complexes.^18^ Evidently, in solution at room temperature, *k*_r_ ≪ ∑*k*_nr_ such that phosphorescence is not observed under these conditions.

## 3 Conclusion

In his extensive review of Pt­(II) complexes with tetradentate ligands,^9^ You noted the paucity of examples that feature 5–5–5 platinacycles (*i.e*., three 5-membered chelate rings), these being limited to the complexes of a dianionic, *N* ^*–*^ *NNN* ^*–*^-coordinating, ethylene-bridged bis­(pyrrole)­diimine and its derivatives.^31^ There appear to be no cyclometalated examples. You speculated that this scarcity may be due to the geometric constraints imposed on π-conjugated ligands if they are to form such platinacycles, which may result in the terminal rings having longer than desirable Pt–L bonds. The isolation of PtL^1^ in the present work is therefore interesting. The Pt–N bonds prove not to be abnormally long, but rather are similar to the lateral Pt–N in Pt­(*NNC*-phbpy)Cl derivatives, for example (phbpyH = 2-phenylbipyridine).^28^ Moreover, PtL^1^ is brightly luminescent, contrary to what would be expected if the ligand field were weak in the event of elongated Pt–N bonds (rendering deactivating d–d excited states thermally accessible). The result might therefore suggest that further work on other ligands that would form 5–5–5 platinacycles may be fruitful in the context of the development of new phosphors. Nevertheless, PtL^1^ shows an unusual propensity to oxidation to the Pt­(III)_2_ dimer Pt_2_L^1^_2_Cl_2_, containing a Pt–Pt bond.

When 8-substituted quinoline rings are introduced in place of the pyridines, the corresponding chelate ring is increased to 6-membered, and the ligand twists, binding in a helical manner such that the complexes are chiral. The nonplanarity of the Pt­(*NCCN*) units probably inhibits the close interfacial approach required to form a Pt­(III) dimer in these cases, and the Pt­(IV) complexes with two axial chloride ligands are favored instead. As frequently observed, the poorer mixing of metal character into the excited states of cyclometalated Pt­(IV) complexes leads to low triplet radiative rate constants. Consequently, while long-lived phosphorescence is observable at 77 K, it cannot compete with fast nonradiative decay processes at room temperature, rendering the complexes nonemissive in solution. The Pt­(II) complexes PtL^2–3^ – formed upon reduction of PtL^2–3^Cl_2_ – are, on the other hand, deep-red-emitting phosphors, albeit with quantum yields that are compromised by the efficient nonradiative decay that is typical in this region of the spectrum.

## 4 Experimental Section

### 4.1 Synthetic Work

Reagents were obtained from commercial sources and used without further purification, unless stated otherwise. All solvents used in the preparative work were at least Analar grade. Dry solvents were obtained from HPLC-grade solvents that had been passed through a Pure Solv 400 solvent purification system and stored over activated 3 or 4 Å molecular sieves. For procedures involving a dry solvent, glassware was oven-dried at 110 °C prior to use. Reactions requiring an inert atmosphere were carried out using Schlenk-line techniques under an atmosphere of nitrogen. Thin-layer chromatography (TLC) was carried out using silica plates (MerckArt 5554) and visualized by UV radiation at 254 and/or 365 nm. NMR spectra were recorded on a Bruker Avance-400 spectrometer. Two-dimensional spectra used to aid assignments (COSY, NOESY, HSQC, and HMBC) were acquired on a Varian VNMRS-600 (600 MHz) or Varian VNMRS-700 (700 MHz) instrument. Chemical shifts (δ) are given in parts per million, referenced to residual protiosolvent resonances, and coupling constants are given in Hertz. Electrospray ionization mass spectral data (positive and negative modes) were obtained on a SQD mass spectrometer interfaced with an Acquity UPLC system with acetonitrile as the carrier solvent. Spectra acquired using an Atmospheric Solids Atomization Probe were recorded on a Waters Xevo QToF mass spectrometer.

The synthetic procedures for all new intermediates, proligands, and complexes are described in the Supporting Information, together with characterization data and ^1^H and ^13^C NMR spectra. The proligand H_2_L^0^ has been reported and characterized previously, synthesized by a nickel-catalyzed homocoupling of ppy-Br. In the present work, it was prepared from the same starting material but through the intermediacy of the boronate and a palladium-catalyzed cross-coupling, as for H_2_L^1^. The ^1^H and ^13^C NMR data for the sample of H^2^L^0^ so prepared were consistent with those in a previous report.

### 4.2 Photophysical Studies

UV–visible absorption spectra were recorded on a Biotek Instruments Uvikon XS spectrometer operated with LabPower software. Emission spectra were acquired on a Jobin Yvon Fluoromax-2 spectrometer equipped with a Hamamatsu R928 photomultiplier tube. All samples were contained within 1 cm path length quartz cuvettes modified for connection to a vacuum line. Degassing was achieved by at least three freeze–pump–thaw cycles while connected to the vacuum manifold: final vapor pressure at 77 K was <5 × 10^–2^ mbar. The emission was recorded at 90° to the excitation source, and spectra were corrected after acquisition for dark count and the spectral response of the detector. The quantum yields were determined relative to an aqueous solution of [Ru­(bpy)_3_]­Cl_3_, for which Φ_lum_ = 0.04.^32^ Emission spectra at 77 K were recorded in 4 mm diameter tubes held within a liquid-nitrogen-cooled quartz Dewar instrument, using the same spectrometer.

Luminescence lifetimes, τ, in solution at 295 K were measured by time-correlated single-photon counting using a pulsed-diode laser as the excitation source (405 nm excitation, pulse length of 60 ps, and repetition rate of 20 kHz or higher for shorter lifetimes). The emission was detected at 90° to the excitation source after passage through a monochromator using an R928 PMT thermoelectrically cooled to −20 °C. The longer lifetimes at 77 K were recorded using the same detector operating in multichannel scaling mode, following excitation with a microsecond pulsed xenon lamp. For all measurements, the decays were much longer than the instrument response, and the data were analyzed by least-squares tail fitting to the following equation:

I(t)=I(0)exp(−kt)+c

where *I*(t) is the intensity of light detected at time *t*, *k* is the first-order rate constant for decay (from which the lifetime τ is obtained through τ = 1/*k*), and *c* is a constant reflecting the intrinsic “dark count” during the measurement. The quality of the fit was assessed by referring to the residuals (difference between the fit and experimental data).

### 4.3 X-ray Crystallography

The X-ray single crystal data have been collected at a temperature of 120.0(2) K using Mo Kα radiation (λ = 0.71073Å) on a Bruker D8Venture with a Photon III MM C7 or C14 CPAD detector, IμS–III-microsource, focusing mirrors diffractometer equipped with a Cryostream (Oxford Cryosystems 700+) open-flow nitrogen cryostat. The structures were solved using Olex2 ^33^ with the ShelXT^34^ structure solution program using Intrinsic Phasing, and refined with the ShelXL^35^ or Olex2.refine^36^ refinement package using least-squares minimization on F^2^. All non-hydrogen atoms were refined with anisotropic displacement parameters. Hydrogen atoms were located on the difference map and refined isotropically on a riding model, unless otherwise specified. Crystal data and parameters of refinement are listed in Tables S1–S3 in the Supporting Information. Crystallographic data for the structures have been deposited with the Cambridge Crystallographic Data Centre with deposition numbers CCDC 2484706–2484712.

### 4.4 Density Functional Theory Calculations

Density functional theory (DFT) and time-dependent density functional theory (TD-DFT) simulations with the Tamm-Dancoff approximation (TDA) were performed on the complexes using the ORCA 5.0.3 quantum chemistry software.^37–39^ Molecular orbital (MO) iso surfaces were visualized using ChimeraX-1.4 ^40^ or Avogadro 1.2.0.^41^ Geometry optimizations of the complexes in the ground state were performed at the B3LYP^42,43^ /def2-SVP^44^ level of theory. Single-point energy (SPE) calculations were performed at the B3LYP/def2-SVP level of theory with the aid of RIJCOSX^45,46^ approximation and using CPCM for CH_2_Cl_2_ in all cases. All calculations were performed using very tight geometry and SCF convergence criteria, and using the atom-pairwise dispersion correction with the Becke-Johnson damping scheme (D3BJ).^47,48^ Frequency calculations confirmed all of the respective optimized geometries to be local minima.

## Supplementary Material



## Footnotes

§ Although there are a few examples of Pt­(II) complexes with tetradentate ligands in which the central chelate is 5-membered, they do not incorporate two *NC*-coordinating units, but typically feature a lateral phenolic or carboxylate oxygen donor.^9^

† Here, and in the Supporting Information, we use an asterisk * to denote intermediates incorporating a *tert*-butyl group.

## Notes

Synthetic and characterization data for proligands and complexes; ^1^H and ^13^C NMR spectra; tables of crystal data and refinement parameters; additional frontier orbital plots; correlation of non-radiative decay of Pt­(II) complexes with excited-state energy (PDF)
