# Supplementary material for: Platinum Complexes of Tetradentate NCCN-Coordinating Ligands: Structures and Photophysical Properties of PtII, Pt2 III and PtIV Compounds
Source: Inorg Chem. 2025 Nov 7;64(46):23000–12. doi: 10.1021/acs.inorgchem.5c04329 (PMC12648668; doi:10.1021/acs.inorgchem.5c04329)
Supplement: Supplementary file 1 [file ic5c04329_si_001.pdf]

Supporting Information

Platinum complexes of tetradentate *NCCN*-coordinating ligands: Structures and photophysical properties of Pt^{II}, Pt^{III}_2 and Pt^{IV} compounds

Yana M. Dikova,[*] Toby J. Blundell, and J. A. Gareth Williams[*]

Department of Chemistry, Durham University, Durham, DH1 3LE, U.K.
yana.dikova@durham.ac.uk   j.a.g.williams@durham.ac.uk

Contents:

Section 1. Synthesis and characterization of proligands and complexes.................. **Page S2**

Section 2. ^{1}H and ^{13}C NMR spectra.. **Page S10**

Section 3. X-ray crystallography: crystal data and structure refinements................. **Page S22**

Section 4. Frontier orbitals and spin density plots .... **Page S25**

Section 5. Non-radiative decay of Pt(II) complexes versus excited-state energy......... **Page S30**

Section 1 Synthesis and characterization of proligands and complexes

(A) Proligands and their precursors

2-(3-Bromo-5-(*tert*-butyl)phenyl)pyridine, ppy*-Br

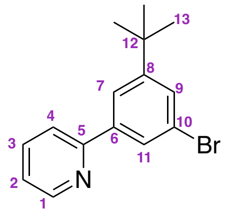

This compound was prepared by a Stille cross-coupling of 1,3-dibromo-5-(*tert*-butyl)benzene (451 mg, 1.54 mmol) and 2-(tributylstannyl)-pyridine (0.5 mL, 569 mg, 1.54 mmol). These two reagents were dissolved in toluene (15 mL) and the solution was degassed by three freeze-pump-thaw cycles. $Pd(PPh_3)_4$ (89 mg, 0.077 mmol) was added under nitrogen and the resulting yellow solution was heated at reflux temperature overnight. After cooling, saturated aqueous KF (5 mL) was added and the mixture was stirred for 30 min. The precipitate was filtered off and the solvent was removed under reduced pressure. The crude product was extracted into DCM (3 × 20 mL), washed with 5% $NaHCO_{3\,(aq)}$ (3 × 15 mL), dried over $MgSO_4$, and purified by gradient column chromatography on silica with hexane / ethyl acetate as the eluant (R_f = 0.6 in hexane : EtOAc = 8:2) to yield a white solid (196 mg, 44% yield). ^{1}H NMR (400 MHz, $CDCl_3$) δ_H / ppm = 8.72 (ddd, J = 5.0, 2.0, 1.0 Hz, 1H), 7.99 – 7.92 (m, 2H), 7.79 (ddd, J = 8.0, 7.5, 2.0 Hz, 1H), 7.76 – 7.69 (m, 1H), 7.60 – 7.55 (m, 1H), 7.32 – 7.23 (m, 1H), 1.40 (s, 9H).

8-(3-Bromo-5-(*tert*-butyl)phenyl)quinoline, pqu*-Br

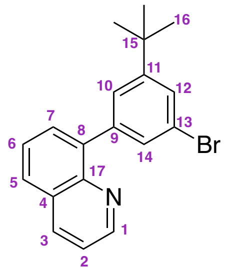

This compound was prepared by Suzuki cross-coupling of 8-quinolinylboronic acid (300 mg, 1.73 mmol) and 1,3-dibromo-5-(*tert*-butyl)benzene (506 mg, 1.73 mmol). A mixture of these two reagents, together with Na_2CO_3 (1.470 g, 13.87 mmol), DME (14 mL) and water (14 mL), was degassed by three freeze-pump-thaw cycles. $Pd(PPh_3)_4$ (100 mg, 0.087 mmol) was added under a nitrogen atmosphere and the mixture was heated at reflux temperature for 72 h. Upon cooling, water was added and the crude product was extracted into DCM. The organic phase was dried over $MgSO_4$, filtered, and the solvent was removed under reduced pressure. The crude product was purified by

gradient column chromatography on silica with hexane / ethyl acetate as the eluant, giving a colourless oil (423 mg, 72% yield) (R_f = 0.5 in hexane : EtOAc = 9:1). ^{1}H NMR (700 MHz, $CDCl_3$) δ_H / ppm = 8.99 – 8.92 (m, 1H, H^1), 8.21 (d, J = 8.0 Hz, 1H, H^3), 7.83 – 7.78 (m, 1H, H^5), 7.67 (dd, J = 7.0, 1.5 Hz, 1H, H^7), 7.61 – 7.59 (m, 1H, H^{14}), 7.59 – 7.55 (m, 1H, H^6), 7.54 – 7.52 (m, 1H, H^{10}), 7.52 – 7.50 (m, 1H, H^{12}), 7.41 (dd, J = 8.0, 4.0 Hz, 1H, H^2), 1.30 (s, 9H, H^{16}). ^{13}C NMR (176 MHz, $CDCl_3$) δ_C / ppm = 153.0 C^{11}, 150.1 C^1, 130.7 C^{14}, 128.8, 128.0 C^{12}, 126.6 C^{10}, 122.1, 121.2 C^2, 35.0 C^{15}, 31.3 C^{16}. HRMS (ESI+) m/z = 340.0701, $[M+H]^+$; calcd for $[C_{19}H_{19}NBr]^+$ = 340.0714.

2-(3-(*tert*-Butyl)-5-(4,4,5,5-tetramethyl-1,3,2-dioxaborolan-2-yl)phenyl)pyridine, ppy*-B

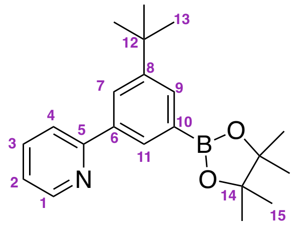

A mixture of ppy*-Br (140 mg, 0.482 mmol), bis(pinacolato)diboron (135 mg, 0.531 mmol) and KOAc (254 mg, 2.895 mmol) were dissolved in 1,4-dioxane (5 mL) and degassed by three freeze-pump-thaw cycles. $PdCl_2(dppf)_2$ (39 mg, 0.048 mmol) was added under nitrogen and the resulting solution was heated at reflux temperature overnight. The solvent was removed under reduced pressure and the crude product was dissolved in DCM, filtered and washed with water, dried over $MgSO_4$, and purified by gradient column chromatography on silica with hexane / ethyl acetate as the mobile phase (R_f = 0.2 in hexane : EtOAc = 9:1) to yield a white solid (129 mg, 92% yield). ^{1}H NMR (700 MHz, $CDCl_3$) δ_H / ppm = 8.67 – 8.62 (m, 1H, H^1), 8.15 – 8.12 (m, 1H, H^7), 8.10 (dd, J = 2.0, 1.0 Hz, 1H, H^{11}), 7.83 (dd, J = 2.0, 1.0 Hz, 1H, H^9), 7.74 (d, J = 8.0 Hz, 1H, H^4), 7.71 – 7.65 (m, 1H, H^3), 7.18 – 7.14 (m, 1H, H^2), 1.35 (s, 9H, H^{13}), 1.29 (s, 12H, H^{15}). ^{13}C NMR (176 MHz, $CDCl_3$) δ_C / ppm = 157.9 C^5, 151.0 C^8, 149.2 C^1, 138.2 C^6, 132.4 C^9, 130.7 C^{11}, 129.2 C^{10}, 127.2 C^7, 122.0 C^2, 121.2 C^4, 83.8 C^{14}, 34.9 C^{12}, 31.5 C^{13}, 24.9 C^{15}. HRMS (ESI^+) m/z = 337.2336 $[M+H]^+$; calcd for $[C_{21}H_{19}NO_2{}^{10}B]^+$ = 337.2328.

8-(3-(*tert*-Butyl)-5-(4,4,5,5-tetramethyl-1,3,2-dioxaborolan-2-yl)phenyl)quinoline, pqu*-B

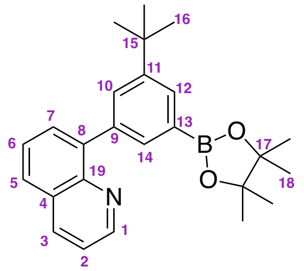

A mixture of 8-(3-bromo-5-(*tert*-butyl)phenyl)quinoline (122 mg, 0.359 mmol), bis(pinacolato)-diboron (100 mg, 0.394 mmol) and KOAc (211 mg, 2.151 mmol) were dissolved in 1,4-dioxane (5 mL) and degassed by three freeze-pump-thaw cycles. $PdCl_2(dppf)_2$ (29 mg, 0.036 mmol) was added under nitrogen and the resulting solution was heated at reflux temperature overnight. The solvent was removed under reduced pressure, and the crude product was dissolved in DCM, filtered and washed with water, dried over $MgSO_4$, and purified by gradient column chromatography on silica with hexane / ethyl acetate as the mobile phase (R_f = 0.2 in hexane : EtOAc = 9:1) to yield a white solid (86 mg, 62% yield). ^{1}H NMR (700 MHz, $CDCl_3$) δ_H / ppm = 9.04 (s, 1H, H^1), 8.30 – 8.24 (m, 1H, H^3), 7.96 – 7.89 (m, 2H, H^{10} and H^{14}), 7.85 (d, J = 8.0 Hz, 1H, H^5), 7.83 (s, 1H, H^{12}), 7.79 (d, J = 7.0 Hz, 1H, H^7), 7.66 – 7.59 (m, 1H, H^6), 7.47 (s, 1H, H^2), 1.44 (d, J = 1.0 Hz, 9H, H^{16}), 1.37 (s, 12H, H^{18}). ^{13}C NMR (176 MHz, $CDCl_3$) δ_C / ppm = 150.0 C^1, 149.8 C^{11}, 134.2 C^{12} and C^{14}, 131.0 C^{10}, 128.7, 127.3 C^5, 126.5 C^6, 120.9 C^2, 83.6 C^{17}, 34.8 C^{13}, 31.6 C^{16}, 24.9 C^{18}. HRMS (ESI$^+$) m/z = 387.2504 $[M+H]^+$; calcd for $[C_{25}H_{31}NO_2{}^{10}B]^+$ = 387.2484.

2,2'-(5,5'-Di-*tert*-butyl-[1,1'-biphenyl]-3,3'-diyl)dipyridine, H_2L^1

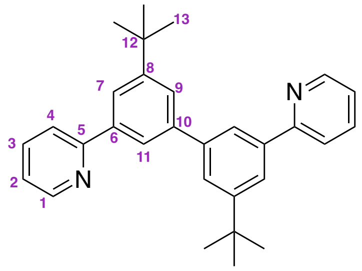

This compound was prepared by Suzuki cross-coupling, using a procedure similar to that described for pqu*-Br above, starting from ppy*-B (150 mg, 0.445 mmol), ppy*-Br (129 mg, 0.445 mmol), Na_2CO_3 (337 mg, 3.56 mmol), $Pd(PPh_3)_4$ (26 mg, 0.022 mmol), DME (3.5 mL) and water (3.5 mL). The product was obtained as a colourless oil (174 mg, 93% yield) (R_f = 0.3 in hexane : EtOAc = 8:2). ^{1}H NMR (400 MHz, $CDCl_3$) δ_H / ppm = 8.78 – 8.74 (m, 1H, H^1), 8.09 – 8.06 (m, 1H, H^{11}), 8.06 – 8.02 (m, 1H, H^7), 7.85 – 7.76 (m, 2H, H^3 and H^4), 7.76 – 7.71 (m, 1H, H^9), 7.32 – 7.24 (m, 1H, H^2), 1.49 (s, 9H, H^{13}) ^{13}C NMR (176 MHz, $CDCl_3$). δ_C / ppm = 157.1 C^5, 152.6, 148.0 C^1, 142.1, 138.6,

126.2 C[9], 124.0 C[11], 123.6 C[7], 122.5 C[2], 122.0 C[4], 35.2 C[12], 30.9 C[13]. HRMS (ESI$^+$) *m/z* = 421.2639 $[M+H]^+$; calcd for $[C_{30}H_{33}N_2]^+$ = 421.2644.

8-(3',5-Di-*tert*-butyl-5'-(pyridin-2-yl)-[1,1'-biphenyl]-3-yl)quinoline, H_2L^2

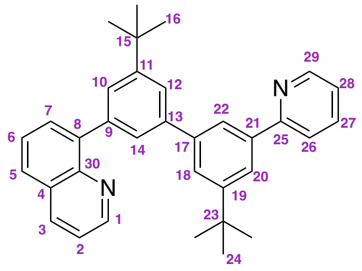

This compound was prepared by Suzuki cross-coupling, using a procedure similar to that described for pqu*-Br above, starting from ppy*-B (72 mg, 0.21 mmol), pqu*-Br (73 mg, 0.21 mmol), Na_2CO_3 (181 mg, 1.71 mmol), $Pd(PPh_3)_4$ (12 mg, 0.011 mmol), DME (2 mL) and water (2 mL). The product was obtained as a white solid (89 mg, 89% yield) (R_f = 0.2 in hexane : EtOAc = 9:1). ^{1}H NMR (700 MHz, $CDCl_3$) δ_H / ppm = 8.89 (dd, J = 4.0, 2.0 Hz, 1H, H[1]), 8.67 – 8.65 (m, 1H, H[29]), 8.15 (dd, *J* = 8.5, 2.0 Hz, 1H, H[3]), 8.00 – 7.96 (m, 1H, H[20]), 7.94 – 7.90 (m, 1H, H[22]), 7.78 (dd, *J* = 8.0, 1.5 Hz, 1H, H[5]), 7.75 (dd, *J* = 7.0, 1.5 Hz, 1H, H[7]), 7.73 – 7.70 (m, 3H, H[14], H[26] and H[27]), 7.66 – 7.65 (m, 1H, H[18]), 7.68 – 7.67 (m, 1H, H[10]), 7.64 – 7.62 (m, 1H, H[12]), 7.56 (dd, *J* = 8.0, 7.0 Hz, 1H, H[6]), 7.35 (dd, *J* = 8.5, 4.0 Hz, 1H, H[2]), 7.18 (s, 1H, H[28]), 1.39 (s, 9H, H[16]), 1.37 (s, 9H, H[24]). ^{13}C NMR (176 MHz, $CDCl_3$) δ_C / ppm = 158.0 C[25], 152.1 C[19], 151.0 C[11], 150.3 C[1], 149.2 C[29], 146.2 C[30], 142.7 C[13] or C[17], 141.4 C[8], 141.1 C[13] or C[17], 139.5 C[9], 137.2 C[26] or C[27], 136.2 C[3], 130.4 C[7], 128.8 C[4], 127.5 C[5], 127.4 C[14], 127.1 C[10], 126.3 C[6], 126.1 C[18], 123.9 C[12], 123.8 C[22], 123.0 C[20], 122.1 C[28], 121.3 C[26] or C[27], 121.0 C[2], 35.1 C[23], 35.0 C[15], 31.6 C[16], 31.5 C[24]. HRMS (ESI+) *m/z* = 471.2783, $[M+H]^+$; calcd for $[C_{34}H_{35}N_2]^+$ = 471.2800.

8,8'-(5,5'-Di-*tert*-butyl-[1,1'-biphenyl]-3,3'-diyl)diquinoline, H_2L^3

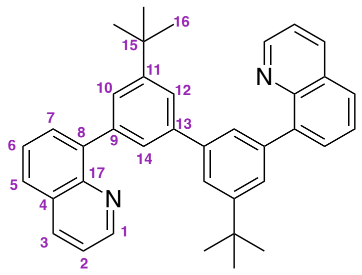

This compound was prepared by Suzuki cross-coupling, using a procedure similar to that described for pqu*-Br above, starting from pqu*-B, pqu*-Br (66 mg, 0.19 mmol), Na_2CO_3 (164 mg, 1.55 mmol), $Pd(PPh_3)_4$ (11 mg, 0.010 mmol), DME (1.5 mL) and water (1.5 mL). The product was obtained as a colourless oil (72 mg, 71% yield) (R_f = 0.5 in hexane : EtOAc = 8:2). ^{1}H NMR (700

MHz, $CDCl_3$) δ_H / ppm = 9.02 – 8.99 (m, 2H, H^{1}), 8.26 (d, J = 8.5 Hz, 2H, H^{3}), 7.84 (ddd, J = 8.7, 7.6, 1.5 Hz, 4H, H^{5} and H^{7}), 7.81 – 7.78 (m, 2H, H^{14}), 7.74 – 7.69 (m, 4H, H^{10} and H^{12}), 7.69 – 7.63 (m, 2H, H^{6}), 7.46 (dd, J = 8.5, 4.0 Hz, 2H, H^{2}), 1.46 (s, 18H, H^{16}). ^{13}C NMR (176 MHz, $CDCl_3$) δ_C / ppm = 150.8 C^{11}, 150.2 C^{1}, 145.8 C^{17}, 141.8 C^{13}, 141.4 C^{4} or C^{8}, 139.1 C^{9}, 136.5 C^{3}, 130.6 C^{7}, 128.8 C^{4} or C^{8}, 127.4 C^{14}, 127.4 C^{5}, 126.8 C^{10} or C^{12}, 126.4 C^{6}, 124.4 C^{10} or C^{12}, 121.0 C^{2}, 35.0 C^{15}, 31.6 C^{16}. HRMS (ESI$^+$) m/z = 521.2957, [M+H]$^+$; calcd for $[C_{38}H_{37}N_2]^+$ = 521.2957.

(B) Platinum complexes

$Pt_2L^1{}_2Cl_2$

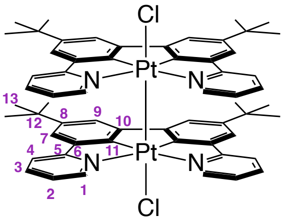

A mixture of K_2PtCl_4 (87 mg, 0.21 mmol) and H_2L^1 (77 mg, 0.18 mmol) in acetic acid (8 mL) was degassed by three freeze-pump-thaw cycles, and then heated at reflux temperature under a nitrogen atmosphere for 72 h. Upon cooling, water (5 mL) was added, and the resulting precipitate was isolated and washed with water (20 mL). It was dried under reduced pressure and purified by recrystallisation from DCM/Et_2O (101 mg, 85% yield). ^{1}H NMR (700 MHz, CD_2Cl_2) δ_H / ppm = 7.86 (d, J = 5.0 Hz, 1H, H^{1}), 7.56 – 7.51 (m, 1H, H^{3}), 7.35 – 7.31 (m, 1H, H^{4}), 7.24 (d, J = 2.0 Hz, 1H, H^{7}), 7.07 (ddd, J = 7.5, 5.0, 1.5 Hz, 1H, H^{2}), 7.03 (d, J = 2.0 Hz, 1H, H^{9}), 1.41 (s, 9H, H^{13}). ^{13}C NMR (176 MHz, CD_2Cl_2) δ_C / ppm = 162.7, 149.8 C^{1}, 149.3, 147.1 C^{8}, 137.7 C^{3}, 121.8 C^{2}, 119.4 C^{9}, 119.0 C^{4}, 118.8 C^{7}, 34.7 C^{12}, 31.8 C^{13}. See Section 3 for details of this compound in the crystal.

PtL^2Cl_2

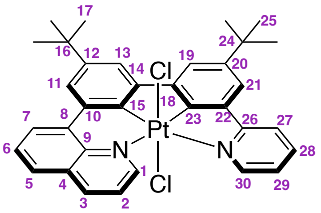

A mixture of K_2PtCl_4 (89 mg, 0.21 mmol) and H_2L^2 (88 mg, 0.19 mmol) in acetic acid (8 mL) was degassed by three freeze-pump-thaw cycles, and then heated at reflux temperature under a nitrogen atmosphere for 72 h. Upon cooling, water (5 mL) was added, and the resulting precipitate was

isolated and washed with water (20 mL). The product was dried under reduced pressure and purified by gradient column chromatography on silica with hexane / ethyl acetate, giving a yellow solid (80 mg, 58% yield). ^{1}H NMR (599 MHz, CD_2Cl_2) δ_H / ppm = 9.69 (dd, J = 5.0, 2.0 Hz, 1H, H^{1}), 8.82 (d, J = 5.5 Hz, 1H, H^{30}), 8.64 (dd, J = 8.0, 2.0 Hz, 1H, H^{3}), 8.53 (dd, J = 8.0, 1.5 Hz, 1H, H^{7}), 8.17 (d, J = 8.0 Hz, 1H, H^{27}), 8.06 – 8.00 (m, 2H, H^{5} and H^{28}), 7.92 – 7.86 (m, 2H, H^{2} and H^{6}), 7.82 (d, J = 2.0 Hz, 1H, H^{11}), 7.75 (d, J = 2.0 Hz, 1H, H^{19}), 7.74 – 7.72 (m, 2H, H^{13} and H^{21}), 7.56 (ddd, J = 8.0, 5.5, 1.5 Hz, 1H, H^{29}), 1.53 (s, 9H, H^{17}), 1.53 (s, 9H, H^{25}). ^{13}C NMR (151 MHz, CD_2Cl_2) δ_C / ppm = 162.7 C^{26}, 152.1 C^{1}, 150.6, 149.7 C^{12}, 149.3 C^{20}, 147.5 C^{30}, 145.8, 144.8, 141.5, 141.2 C^{3}, 139.5 C^{28}, 136.7, 133.0, 132.2 C^{7}, 130.7, 129.6, 129.3 C^{5}, 128.1 C^{6}, 123.6 C^{11}, 123.3 C^{29}, 121.5 C^{2}, 121.2 C^{27}, 120.8 C^{19}, 119.9 C^{13} and C^{21}, 119.8 C^{13} and C^{21}, 34.9 C^{24}, 34.6 C^{16}, 31.6 C^{25}, 31.4 C^{17}. HRMS (ESI^{+}) *m/z* = 733.1619 [M]$^{+}$; calcd for $[C_{34}H_{33}N_2Cl_2{}^{194}Pt]^{+}$ = 733.1648.

PtL^3Cl_2

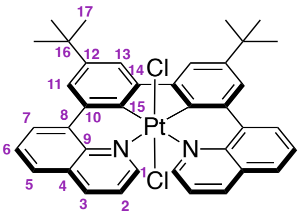

A mixture of K_2PtCl_4 (56 mg, 0.14 mmol) and H_2L^3 (62 mg, 0.12 mmol) in acetic acid (6 mL) was degassed by three freeze-pump-thaw cycles, and then heated at reflux temperature under a nitrogen atmosphere for 72 h. Upon cooling, water (5 mL) was added, and the resulting precipitate was isolated and washed with water (20 mL). The complex was purified by fractional precipitations from DCM / diethyl ether, yielding a yellow solid (39 mg, 42% yield). ^{1}H NMR (599 MHz, $CDCl_3$) δ_H / ppm = 8.82 (dd, $^{3}J_{195Pt\text{-}1H} \approx 14$ Hz, J = 5.0, 1.5 Hz, 1H, H^{1}), 8.52 – 8.46 (m, 2H, H^{3} and H^{7}), 7.92 (dd, J = 8.0, 1.5 Hz, 1H, H^{5}), 7.86 – 7.81 (m, 1H, H^{6}), 7.78 – 7.74 (m, 2H, H^{11} and H^{13}), 7.55 (dd, J = 8.0, 5.0 Hz, 1H, H^{2}), 1.50 (s, 9H, H^{17}). ^{13}C NMR (151 MHz, $CDCl_3$) δ_C / ppm = 153.9 C^{1}, 149.0, 148.4, 142.2 C^{9}, 141.1 C^{3}, 136.8, 132.9, 131.9 C^{7}, 131.1, 129.8 C^{4}, 128.3 C^{5} or C^{6}, 128.2 C^{5} or C^{6}, 123.8 C^{11}, 120.7 C^{2}, 119.4 C^{13}, 34.6 C^{16}, 31.8 C^{17}. HRMS (ESI^{+}) *m/z* = 783.1816 [M]$^{+}$; calcd for $[C_{38}H_{35}N_2Cl_2{}^{194}Pt]^{+}$ = 783.1804.

PtL[1]

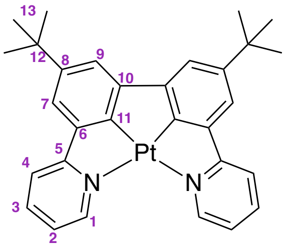

A mixture of PtL^1Cl_2 (10 mg, 0.015 mmol) and activated zinc (20 mg) in dry DCM (3 mL) was degassed by three freeze-pump-thaw cycles. The suspension was heated at reflux temperature under a nitrogen atmosphere for 120 h. Upon cooling, the mixture was filtrated through celite and the solvent was evaporated from the filtrate under reduced pressure to give the product as a red solid (7 mg, 79% yield). ^{1}H NMR (400 MHz, CD_2Cl_2) δ_H / ppm = 8.88 (ddd, $^3J_{195Pt\text{-}1H} \approx 15$ Hz, J = 5.5, 1.5, 1.0 Hz, 1H, H^1), 7.97 (ddd, J = 8.0, 7.0, 1.5 Hz, 1H, H^3), 7.91 (d, J = 8.0, 1.5, 1.0 Hz, 1H, H^4), 7.53 – 7.47 (m, 1H, H^7), 7.47 – 7.39 (m, 2H, H^2 and H^9), 1.47 (s, 9H, H^{13}). ^{13}C NMR (151 MHz, $CDCl_3$) δ_C / ppm = 150.7 C^1, 138.4 C^3, 122.4 C^9, 119.2 C^4, 118.6 C^2, 117.2 C^7, 33.2 C^{13}. HRMS (ESI$^+$) *m/z* = 613.2115 $[M]^+$; calcd for $[C_{30}H_{31}N_2{}^{194}Pt]^+$ = 613.2114.

PtL[2]

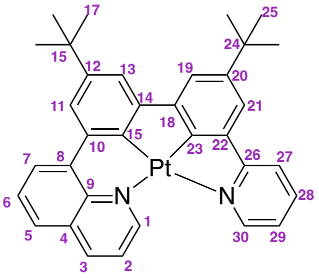

A mixture of PtL^2Cl_2 (10 mg, 0.014 mmol) and activated zinc (20 mg) in dry DCM (3 mL) was degassed by three freeze-pump-thaw cycles. The suspension was heated at reflux temperature under a nitrogen atmosphere for 120 h. Upon cooling, the mixture was filtrated through celite and the solvent was evaporated from the filtrate under reduced pressure to give the product as a red solid (5 mg, 54% yield). ^{1}H NMR (599 MHz, CD_2Cl_2) δ_H / ppm = 9.56 (dd, J = 5.0, 2.0 Hz, 1H, H^1), 8.60 (dd, J = 8.0, 2.0 Hz, 1H, H^3), 8.58 – 8.53 (m, 2H, H^7 and H^{30}), 7.97 – 7.89 (m, 3H, H^5, H^{27} and H^{28}), 7.83 – 7.76 (m, 2H, H^6 and H^{11}), 7.66 (dd, J = 8.0, 5.0 Hz, 1H, H^2), 7.52 (d, J = 2.0 Hz, 1H, H^{13}), 7.49 (d, J = 1.5 Hz, 1H, H^{21}), 7.48 (d, J = 1.5 Hz, 1H, H^{19}), 7.39 (ddd, J = 7.0, 5.5, 1.5 Hz, 1H, H^{29}), 1.49 (s, 9H, H^{17}), 1.47 (s, 9H, H^{25}). ^{13}C NMR (151 MHz, CD_2Cl_2) δ_C / ppm = 167.5 C^{26}, 163.5, 156.6, 152.5 C^1, 152.2, 148.3 C^{30}, 147.2 C^{12}, 146.3 C^{20}, 145.1, 141.3, 141.2, 139.7 C3, 139.4, 138.1 C^{28}, 134.0, 130.7, 130.7 C^7, 127.8, 127.5 C^6, 122.0 C^{29}, 120.7 C^2, 120.5 C^{11}, 119.3 C^{27}, 117.7 C^{19}, 117.1

C^{13}, 116.9 C^{21}, 34.9 C^{24}, 34.7 C^{24}, 31.5 C^{25}, 31.4 C^{17}. HRMS (ASAP$^+$) *m/z* = 662.2202 $[M]^+$; calcd for $[C_{34}H_{32}N_2{}^{194}Pt]^+$ = 662.2192.

PtL^3

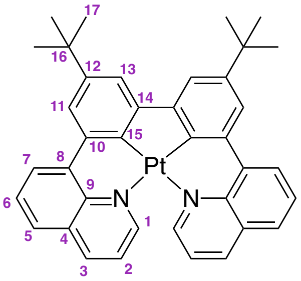

A mixture of PtL^3Cl_2 (10 mg, 0.013 mmol) and activated zinc (20 mg) in dry DCM (3 mL) was degassed by three freeze-pump-thaw cycles. The suspension was heated at reflux temperature under a nitrogen atmosphere for 120 h. Upon cooling, the mixture was filtrated through celite and the solvent was evaporated from the filtrate under reduced pressure to give the product as a red solid (6 mg, 65% yield). ^{1}H NMR (599 MHz, CD_2Cl_2) δH / ppm 8.65 (d, *J* = 8.0 Hz, 2H, H^7), 8.56 (dd, *J* = 8.0, 1.5 Hz, 2H, H^3), 8.36 (dd, $^3J_{195Pt\text{-}1H} \approx$ 19 Hz, *J* = 5.0, 1.5 Hz, 2H, H^1), 7.92 (d, *J* = 8.0 Hz, 2H, H^5), 7.87 – 7.80 (m, 4H, H^6 and H^{11}), 7.61 (d, *J* = 2.0 Hz, 2H, H^{13}), 7.44 (dd, J = 8.0, 5.0 Hz, 2H, H^2), 1.50 (s, 18H, H^{17}). ^{13}C NMR (151 MHz, CD_2Cl_2) δC / ppm 155.4 C^{15}, 153.7 C^1, 147.3 C^{14}, 147.0 C^{12}, 141.7 C^9, 139.2 C^8, 138.8 C^3, 133.3 C^{10}, 129.7 C^7, 129.7 C^4, 127.5 C^6, 127.2 C^5, 121.0 C^2, 120.2 C^{11}, 116.3 C^{13}, 34.6 C^{16}, 31.4 C^{17}. HRMS (ASAP$^+$) *m/z* = 713.2420 $[M]^+$; calcd for $[C_{38}H_{35}N_2{}^{194}Pt]^+$ = 713.2427.

Section 2 ^{1}H and ^{13}C NMR spectra

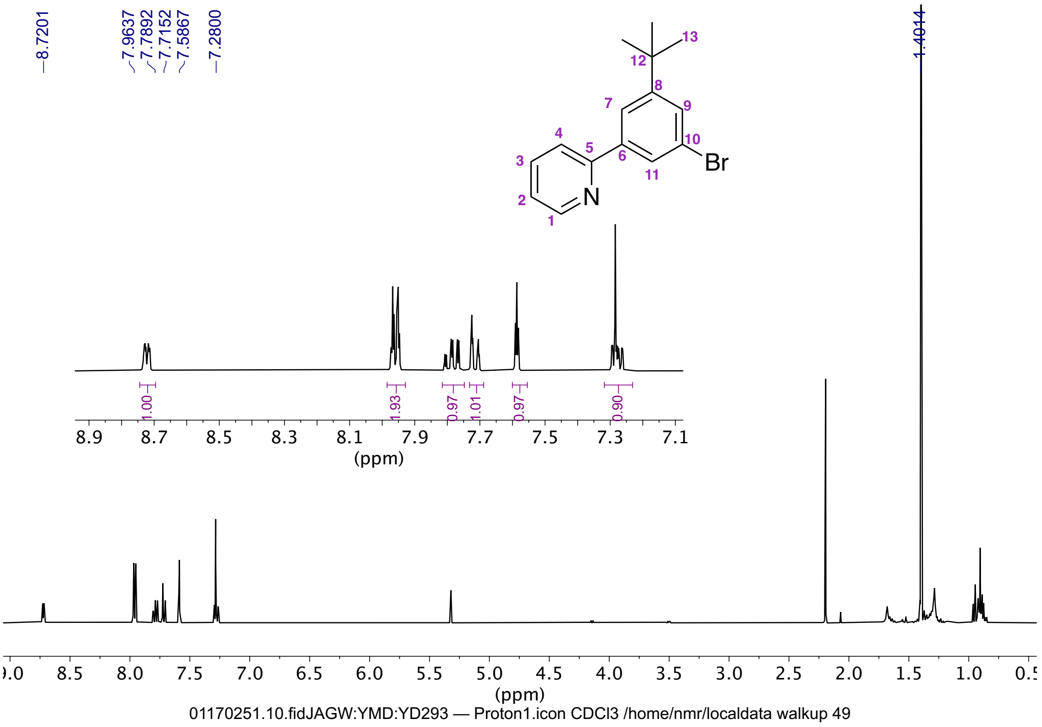

Figure S1. *^{1}H NMR spectrum of ppy*-Br in CDCl$_3$*

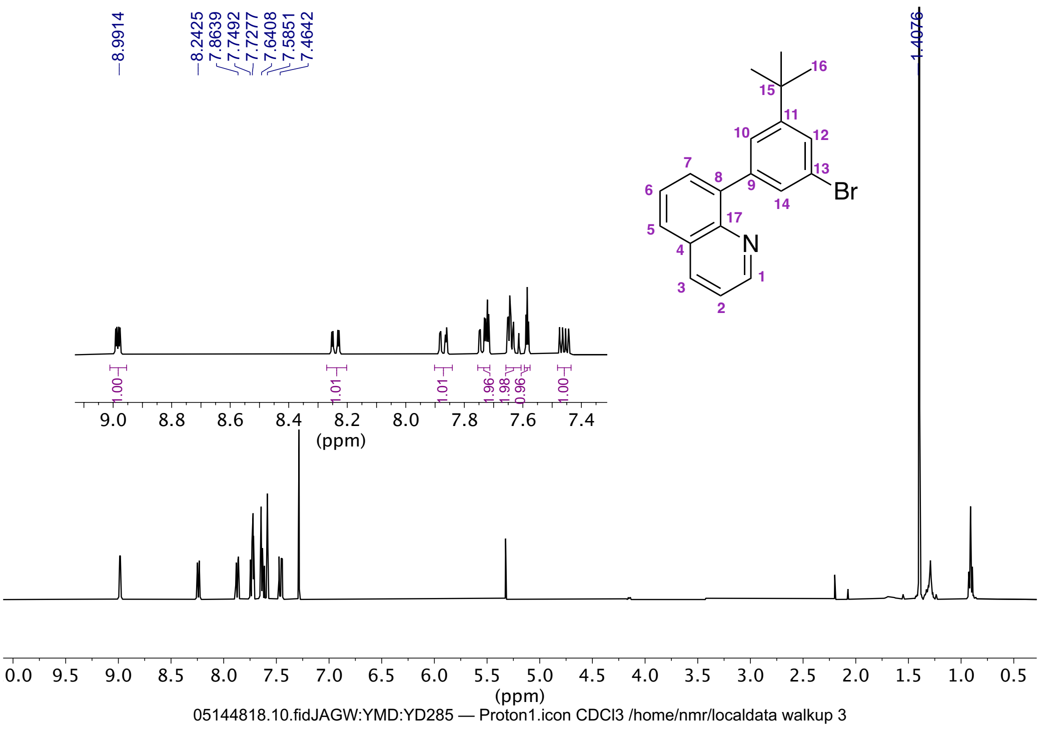

Figure S2. *^{1}H NMR spectrum of pqu*-Br in CDCl$_3$*

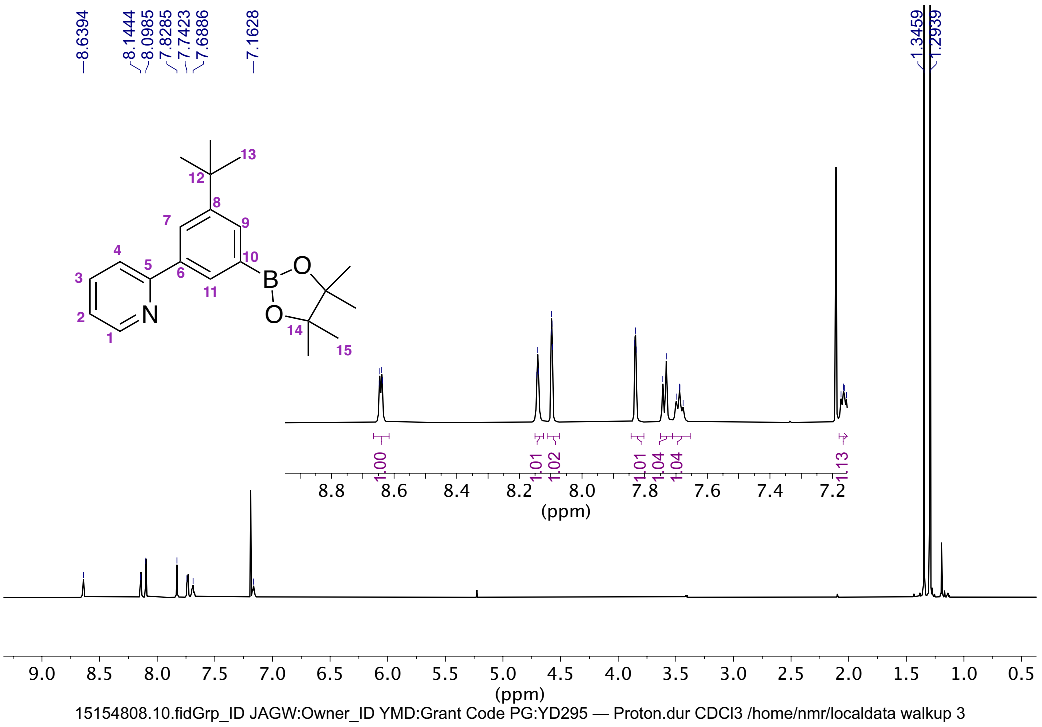

Figure S3. *^{1}H NMR spectrum of ppy*-B in $CDCl_3$*

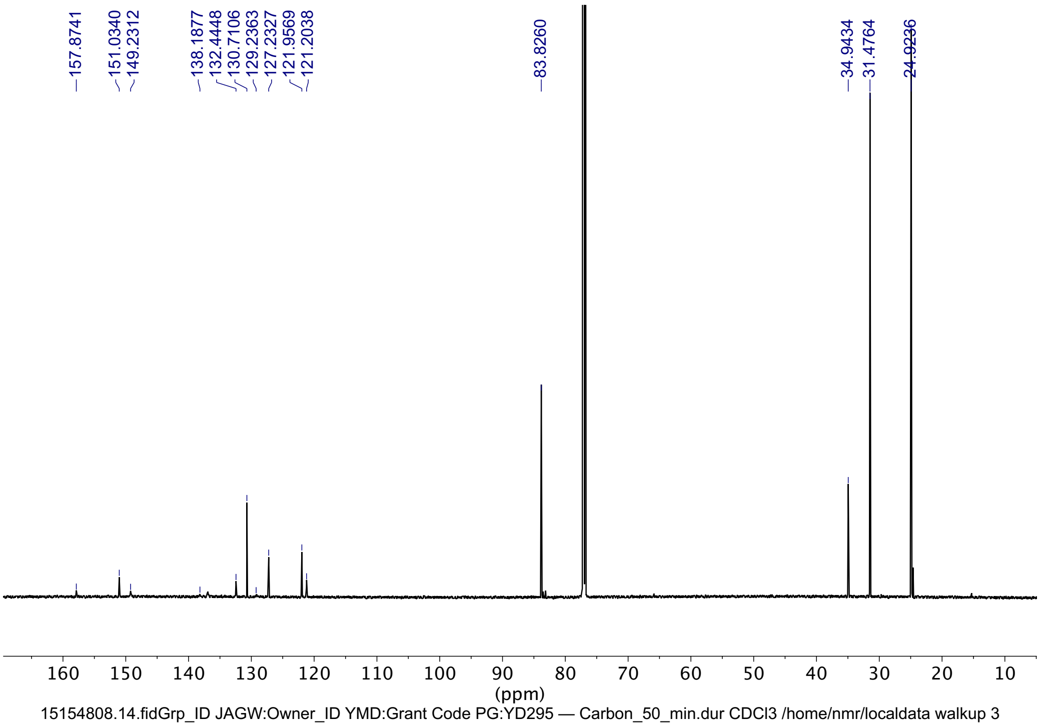

Figure S4. *^{13}C NMR spectrum of ppy*-B in $CDCl_3$*

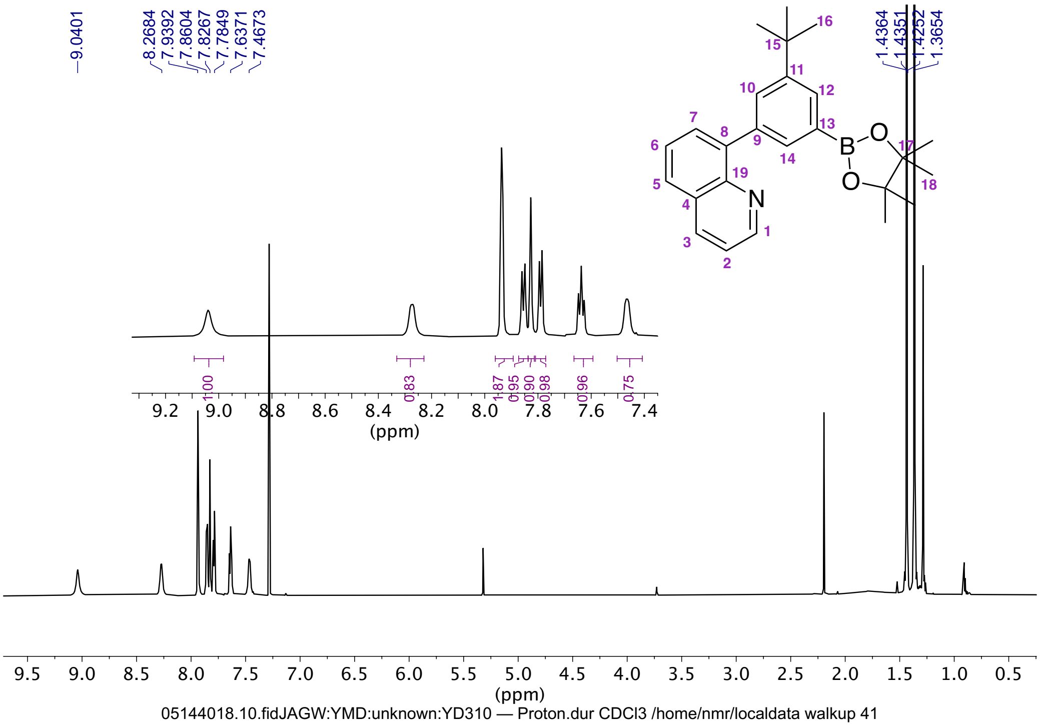

Figure S5. *^{1}H NMR spectrum of pqu*-B in $CDCl_3$*

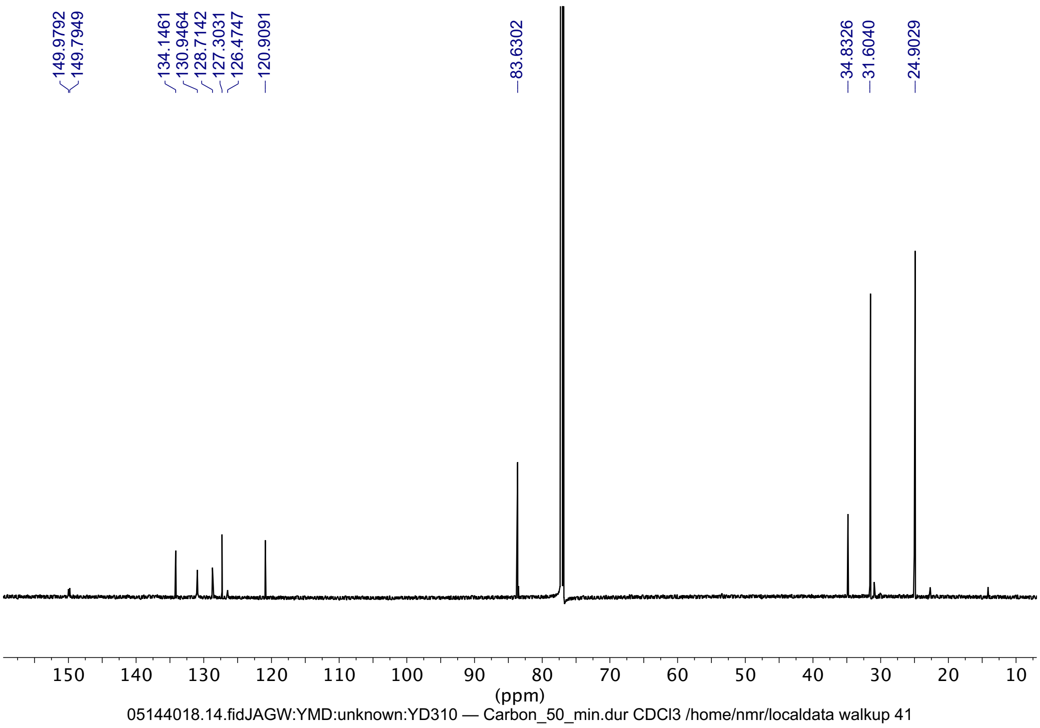

Figure S6. *^{13}C NMR spectrum of pqu*-B in $CDCl_3$*

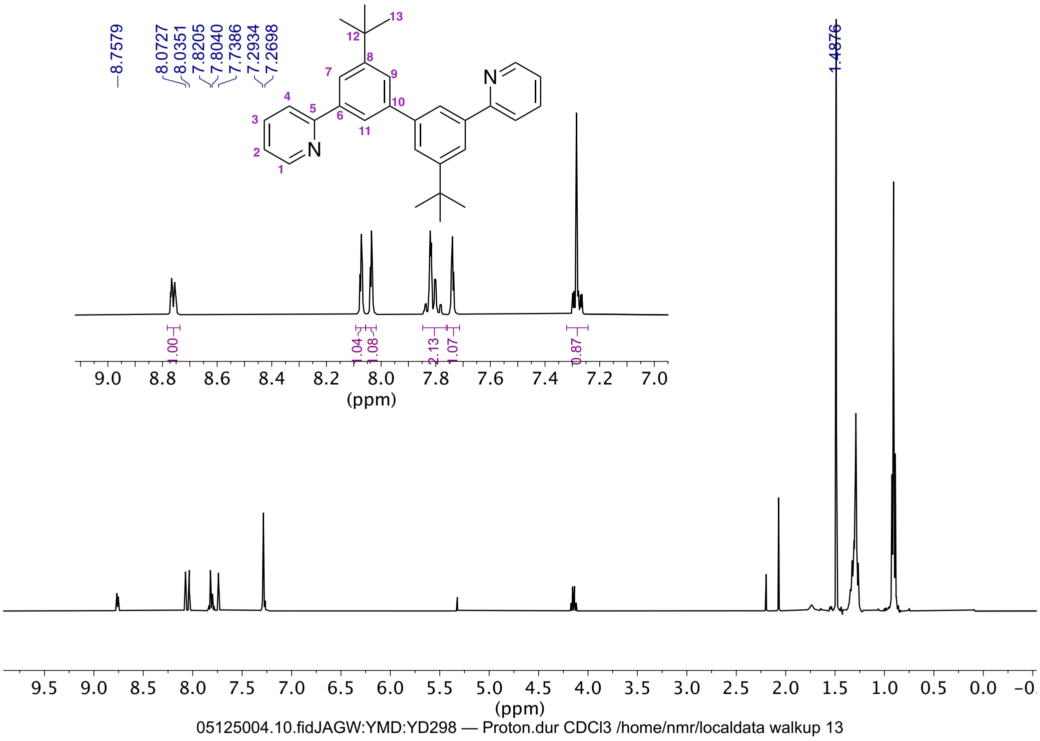

Figure S7. *^{1}H NMR spectrum of H_2L^1 in $CDCl_3$*

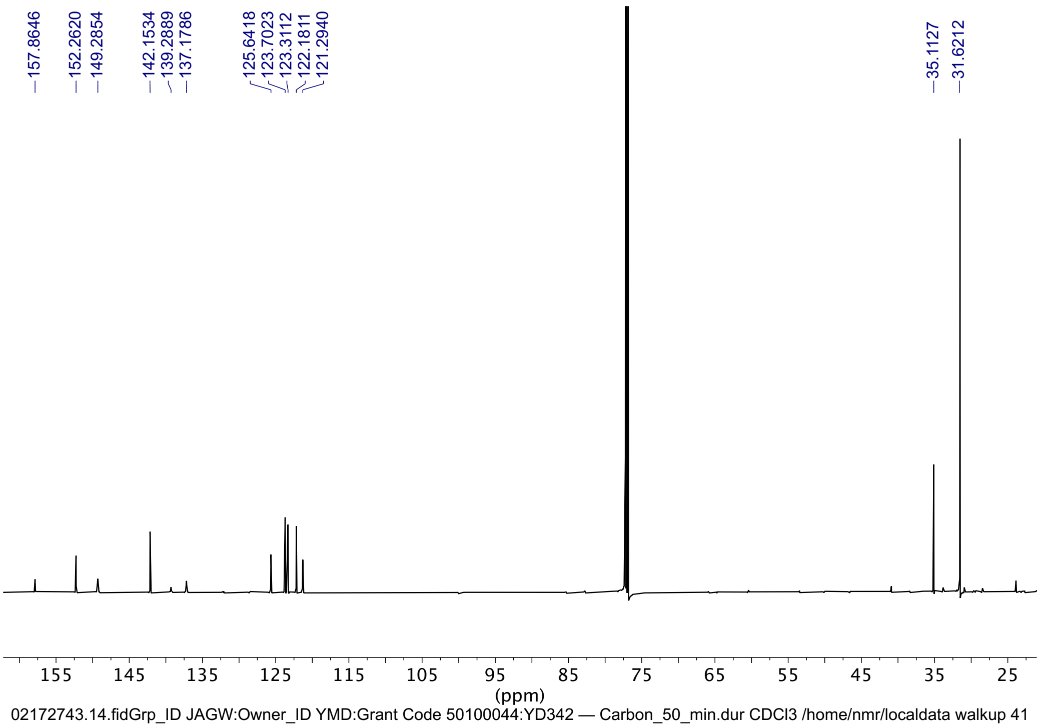

Figure S8. *^{13}C NMR spectrum of H_2L^1 in $CDCl_3$*

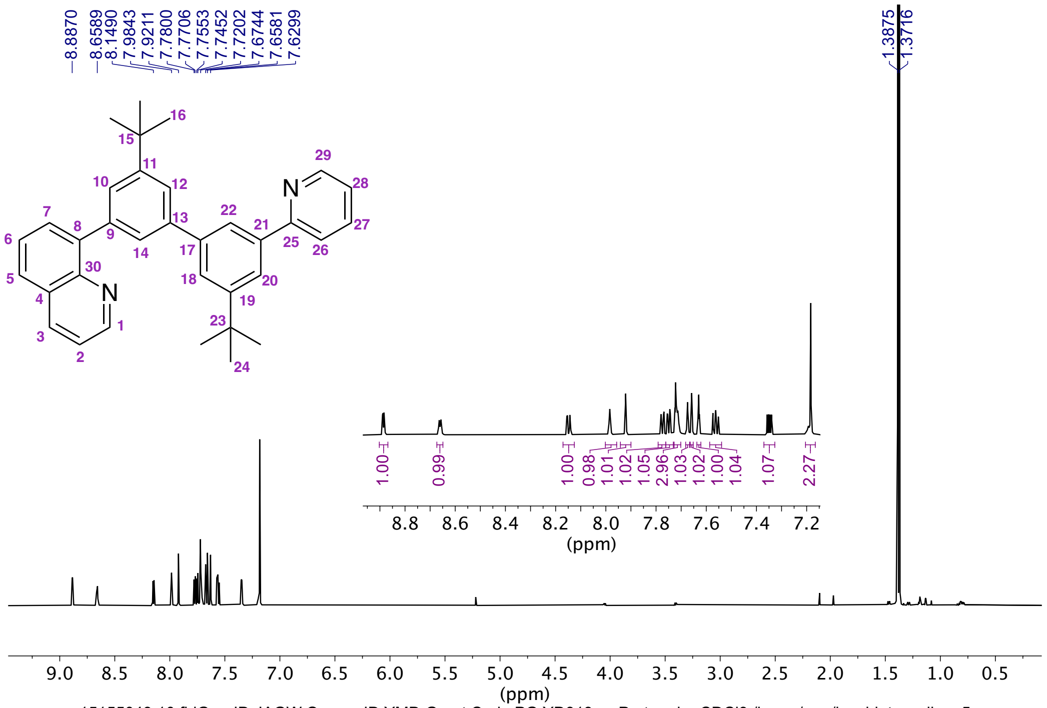

15155049.10.fidGrp_ID JAGW:Owner_ID YMD:Grant Code PG:YD313 — Proton.dur CDCl3 /home/nmr/localdata walkup 5

Figure S9. *^{1}H NMR spectrum of H_2L^2 in $CDCl_3$*

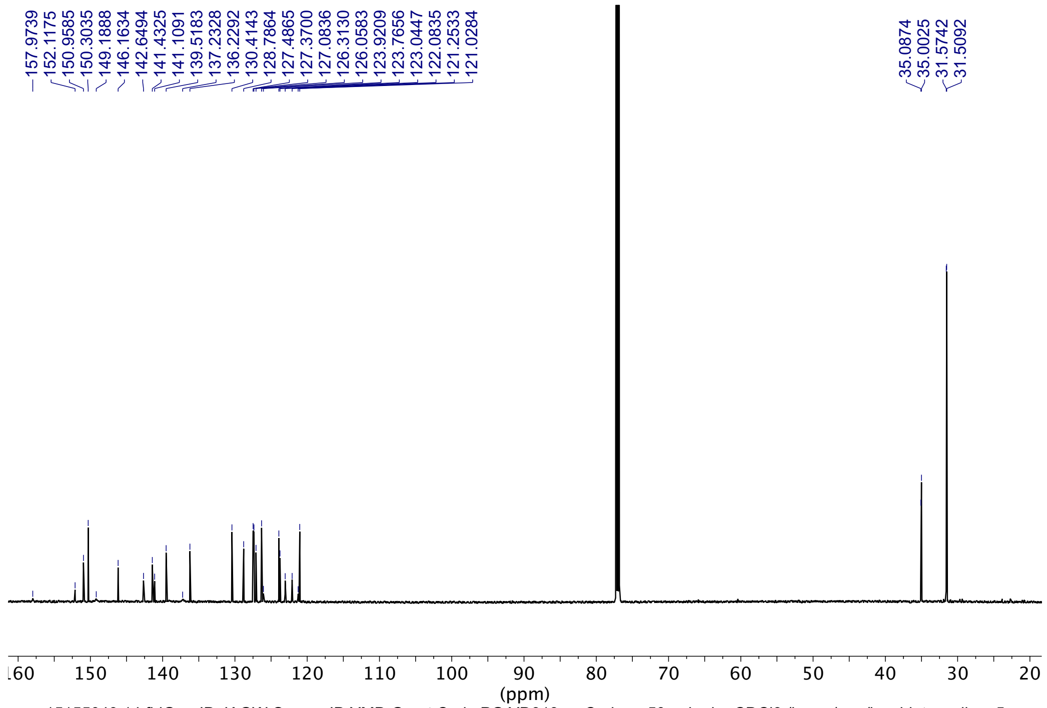

15155049.14.fidGrp_ID JAGW:Owner_ID YMD:Grant Code PG:YD313 — Carbon_50_min.dur CDCl3 /home/nmr/localdata walkup 5

Figure S10. *^{13}C NMR spectrum of H_2L^2 in $CDCl_3$*

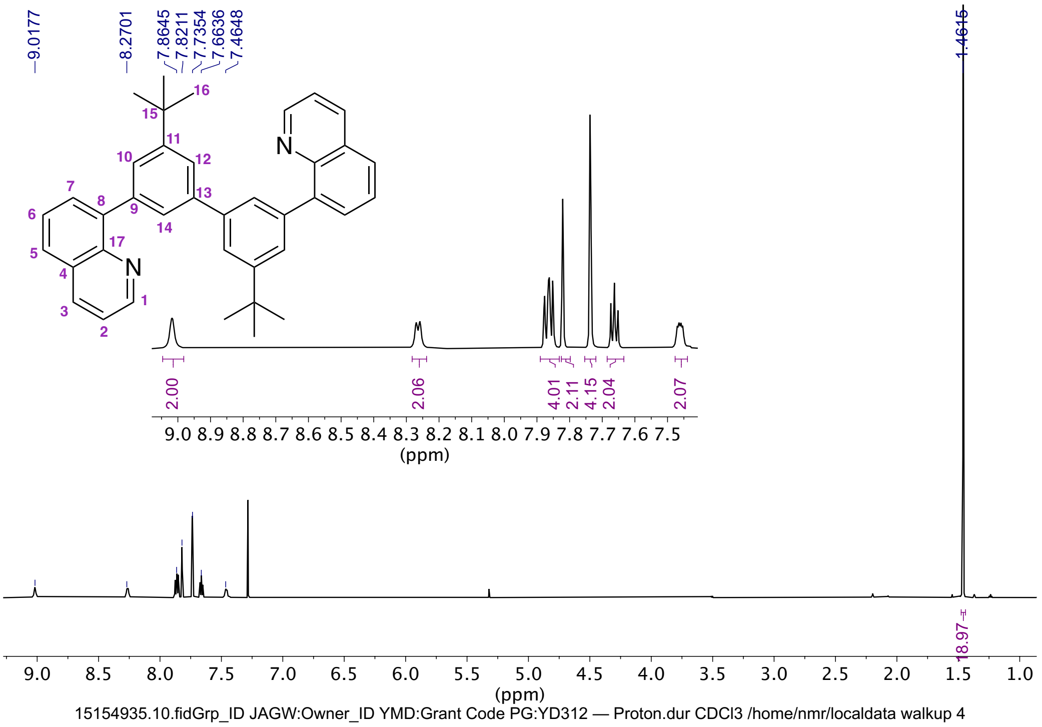

Figure S11. ^{1}H NMR spectrum of H_2L^3 in $CDCl_3$

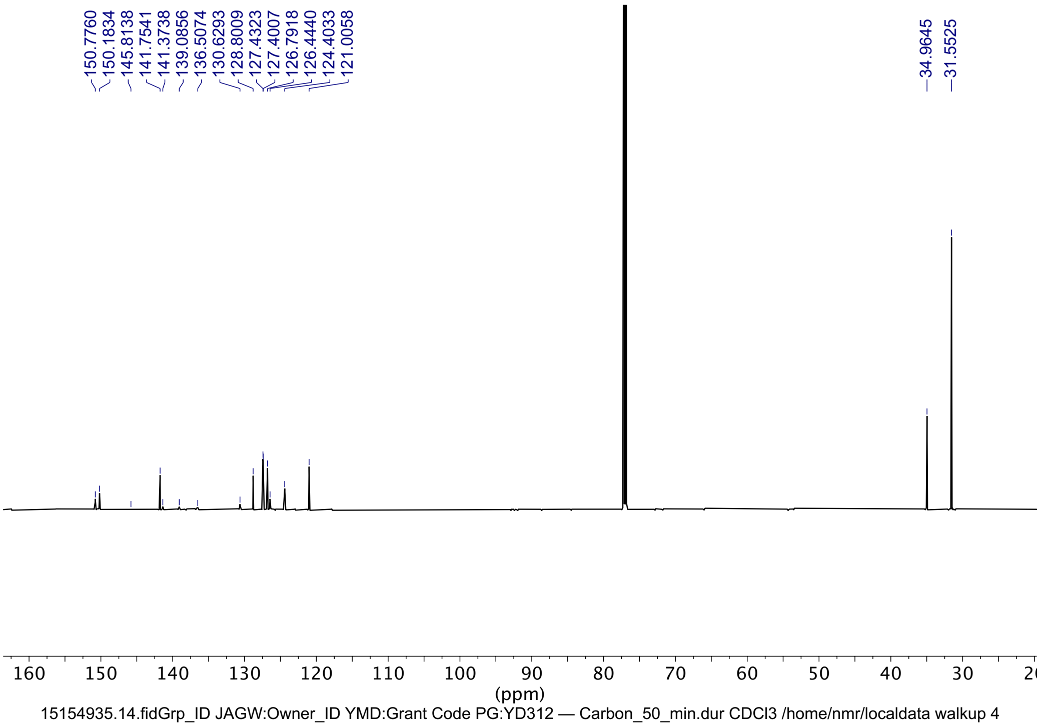

Figure S12. ^{13}C NMR spectrum of H_2L^3 in $CDCl_3$

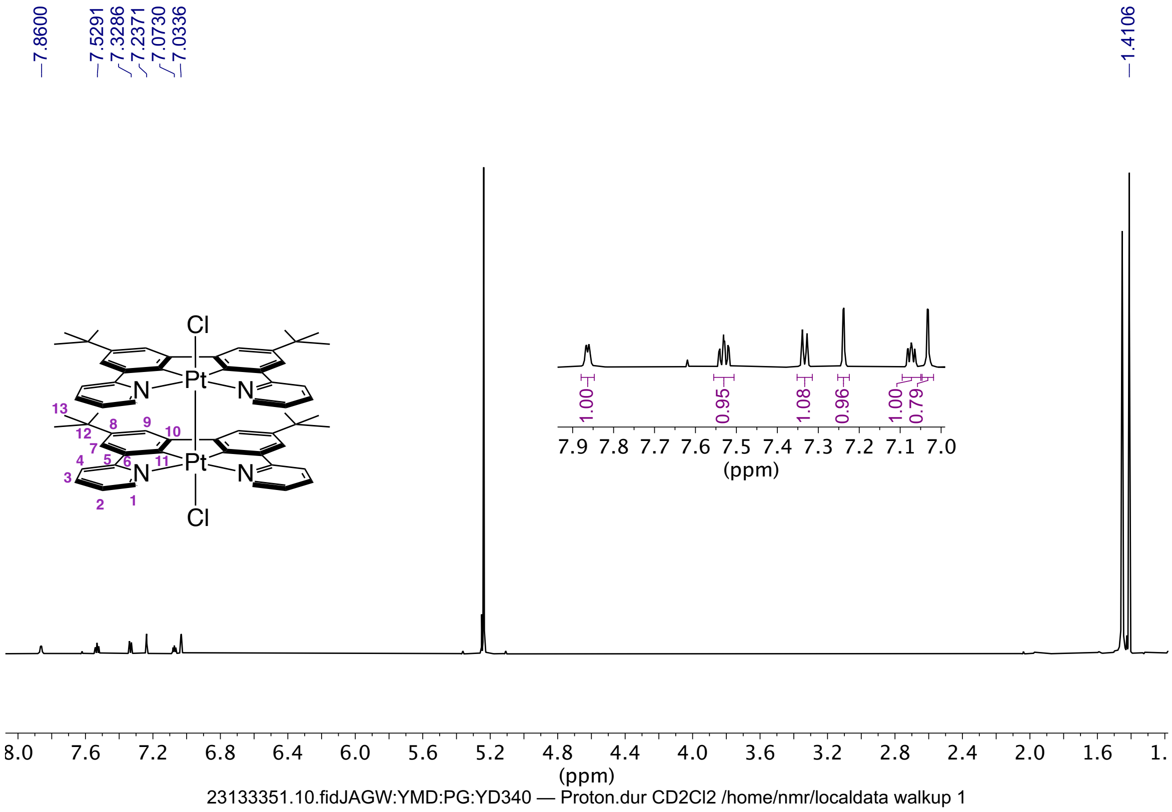

Figure S13. *^{1}H NMR spectrum of $Pt_2L^1{}_2Cl_2$ in CD_2Cl_2*

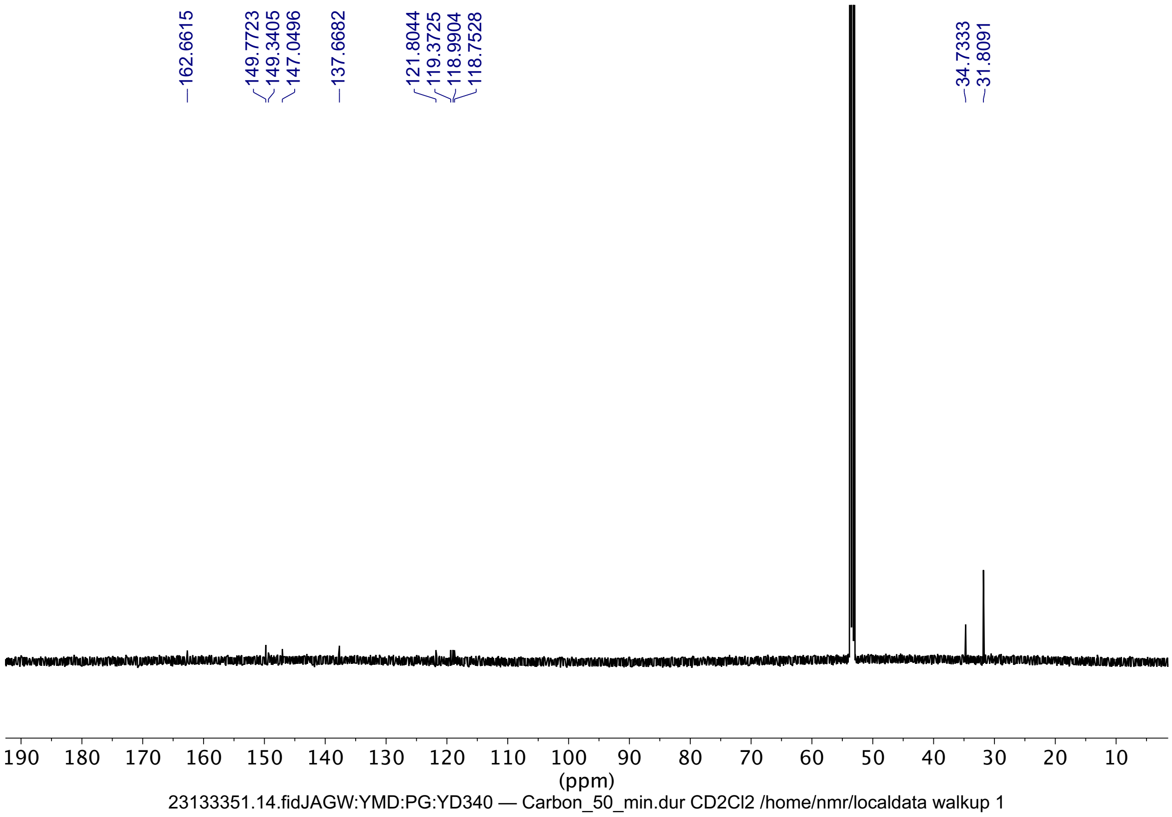

Figure S14. *^{13}C NMR spectrum of $Pt_2L^1{}_2Cl_2$ in CD_2Cl_2*

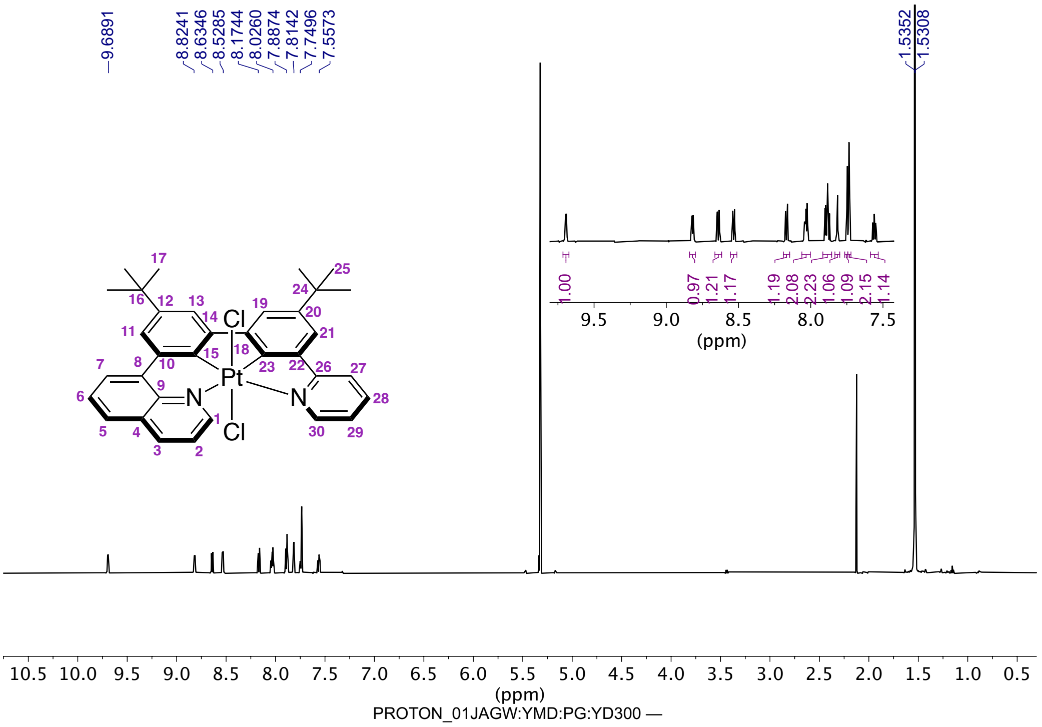

Figure S15. *^{1}H NMR spectrum of $PtL^{2}Cl_{2}$ in $CD_{2}Cl_{2}$*

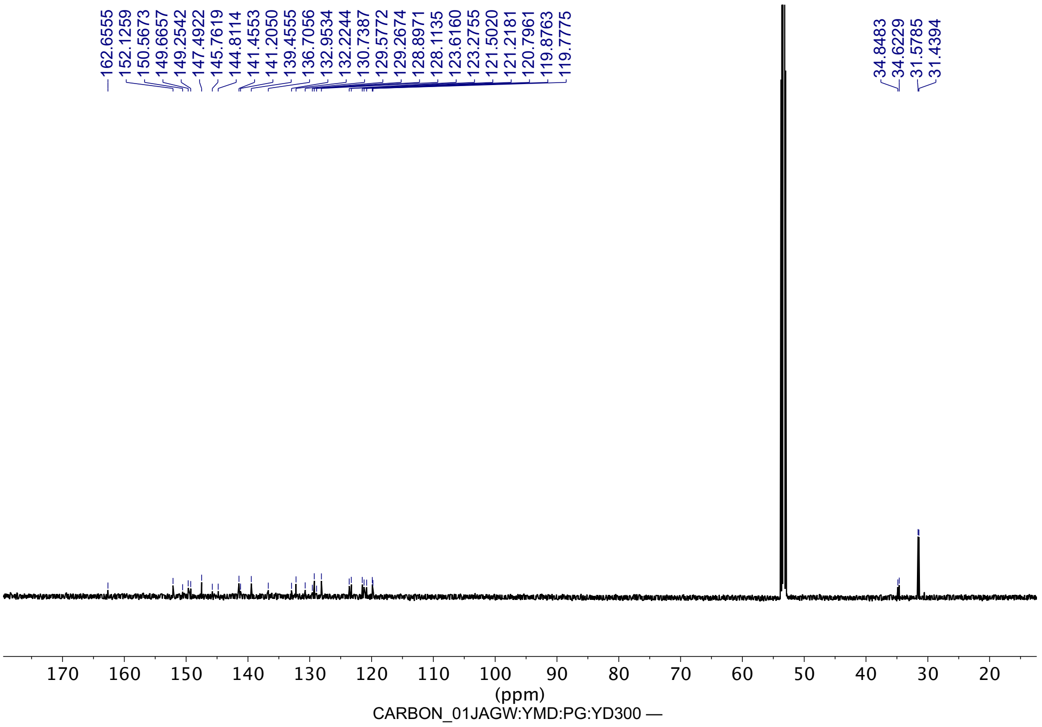

Figure S16. *^{13}C NMR spectrum of $PtL^{2}Cl_{2}$ in $CD_{2}Cl_{2}$*

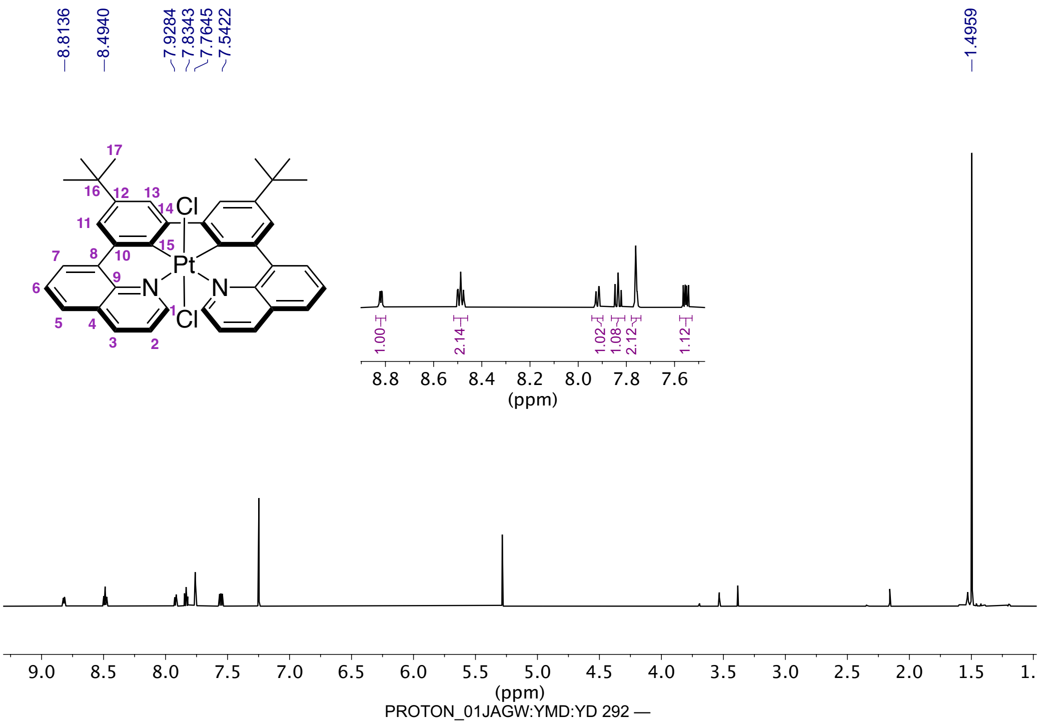

Figure S17. *^{1}H NMR spectrum of $PtL^{3}Cl_{2}$ in $CDCl_{3}$*

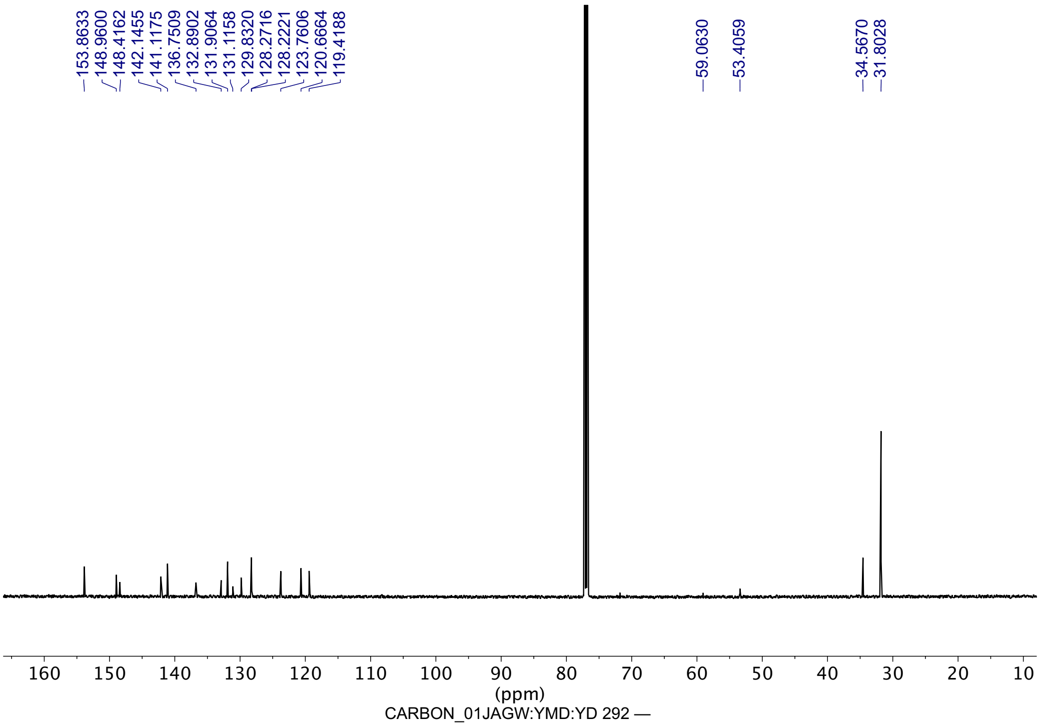

Figure S18. *^{13}C NMR spectrum of $PtL^{3}Cl_{2}$ in $CDCl_{3}$*

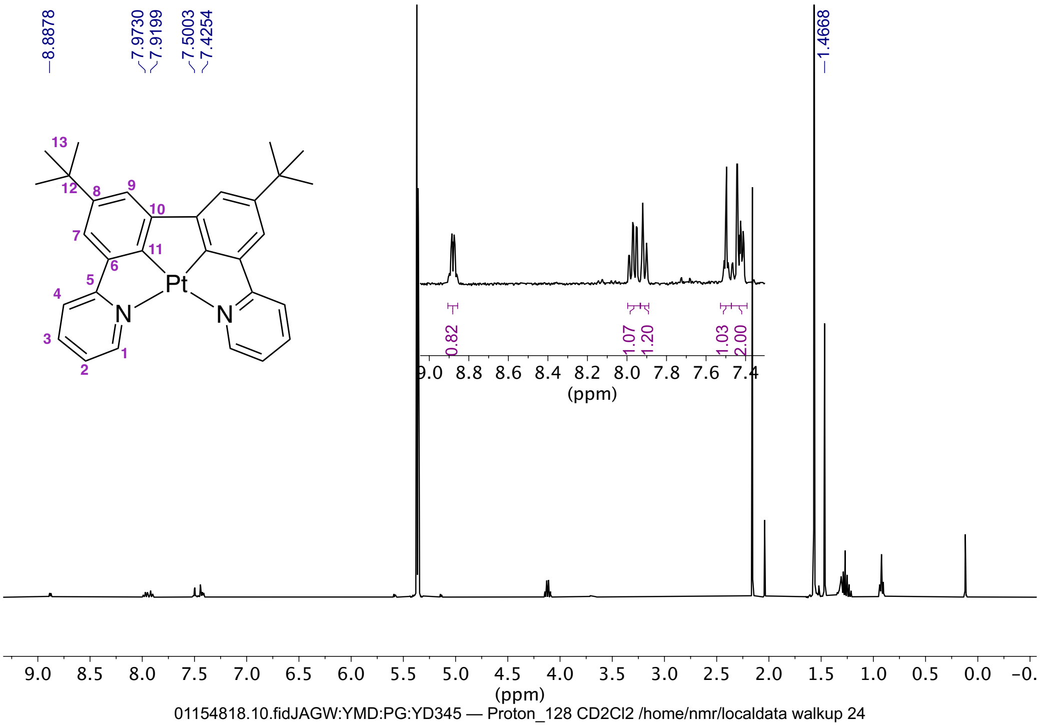

Figure S19. *^{1}H NMR spectrum of PtL^{1} in CD_2Cl_2*

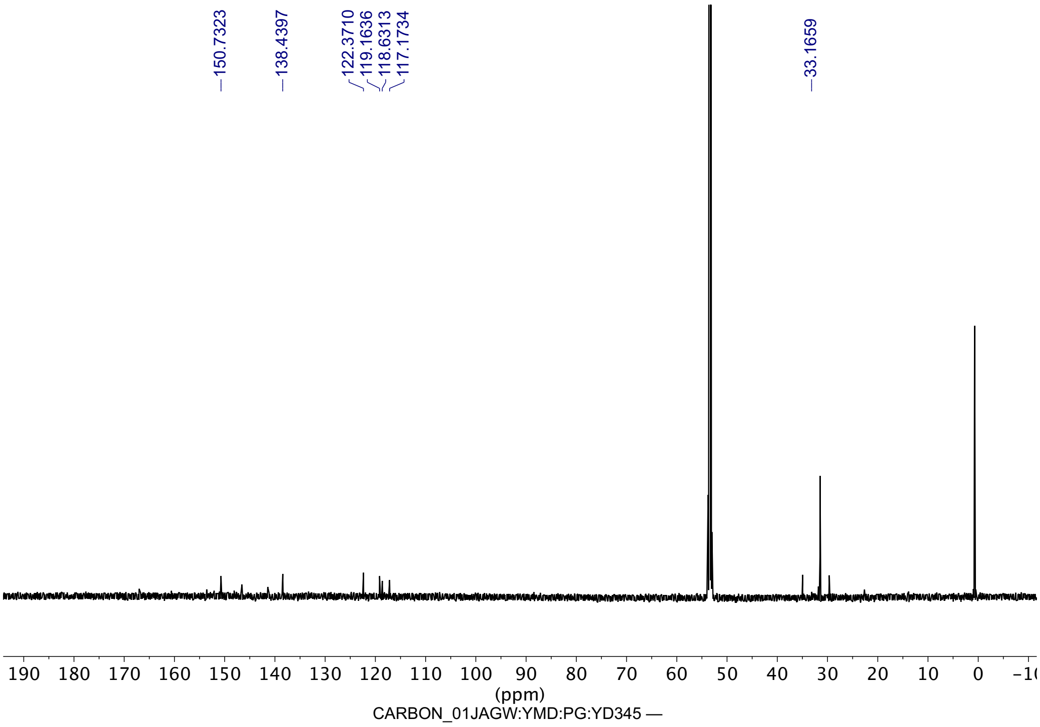

Figure S20. *^{13}C NMR spectrum of PtL^{1} in CD_2Cl_2*

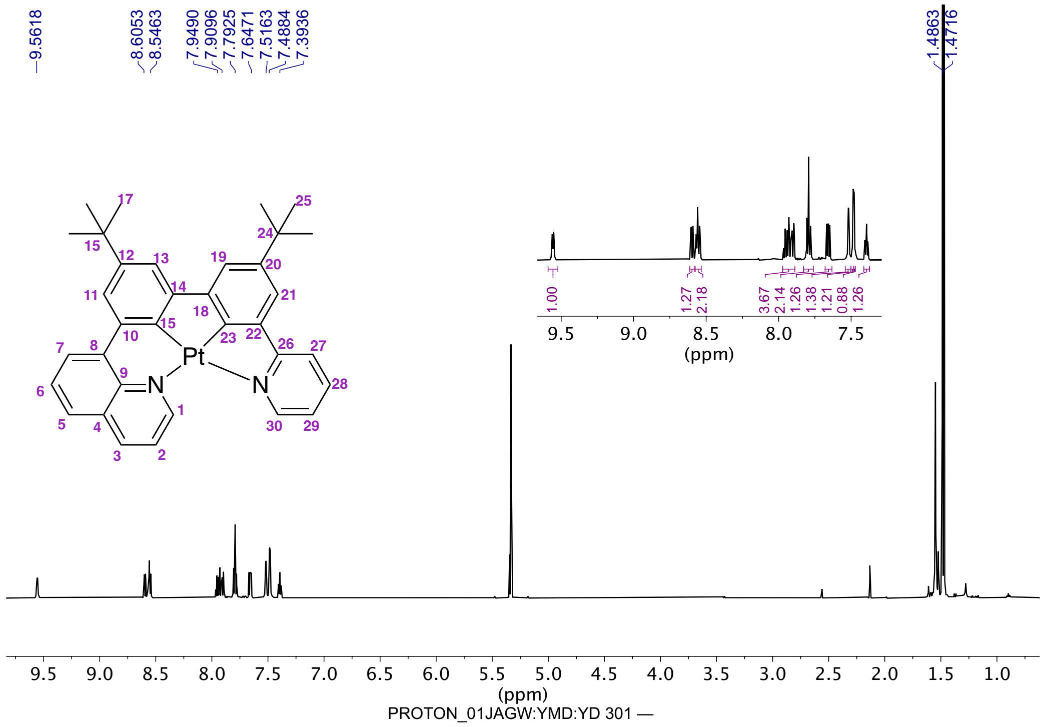

Figure S21. *^{1}H NMR spectrum of PtL^{2} in CD_2Cl_2*

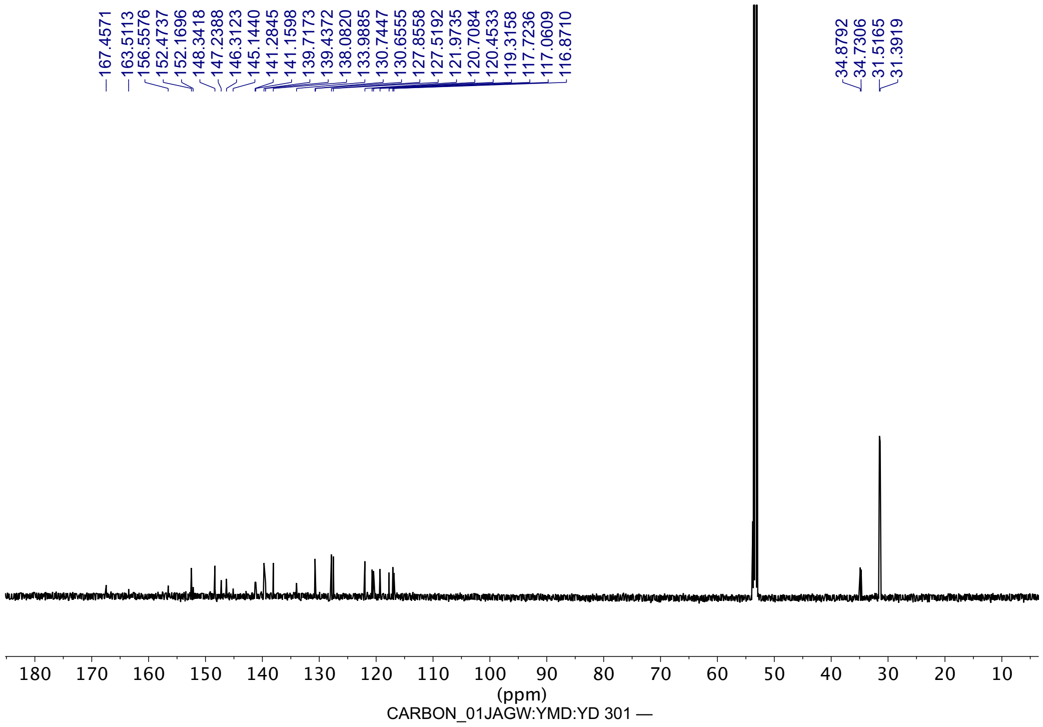

Figure S22. *^{13}C NMR spectrum of PtL^{2} in CD_2Cl_2*

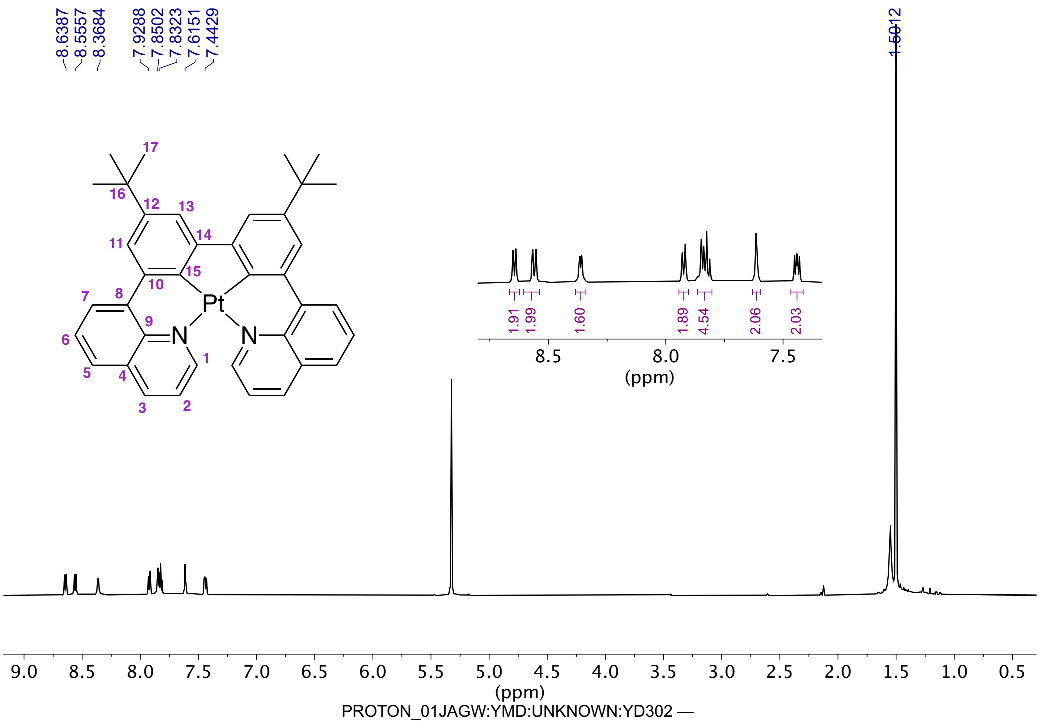

Figure S23. *^{1}H NMR spectrum of PtL^{3} in CD_2Cl_2*

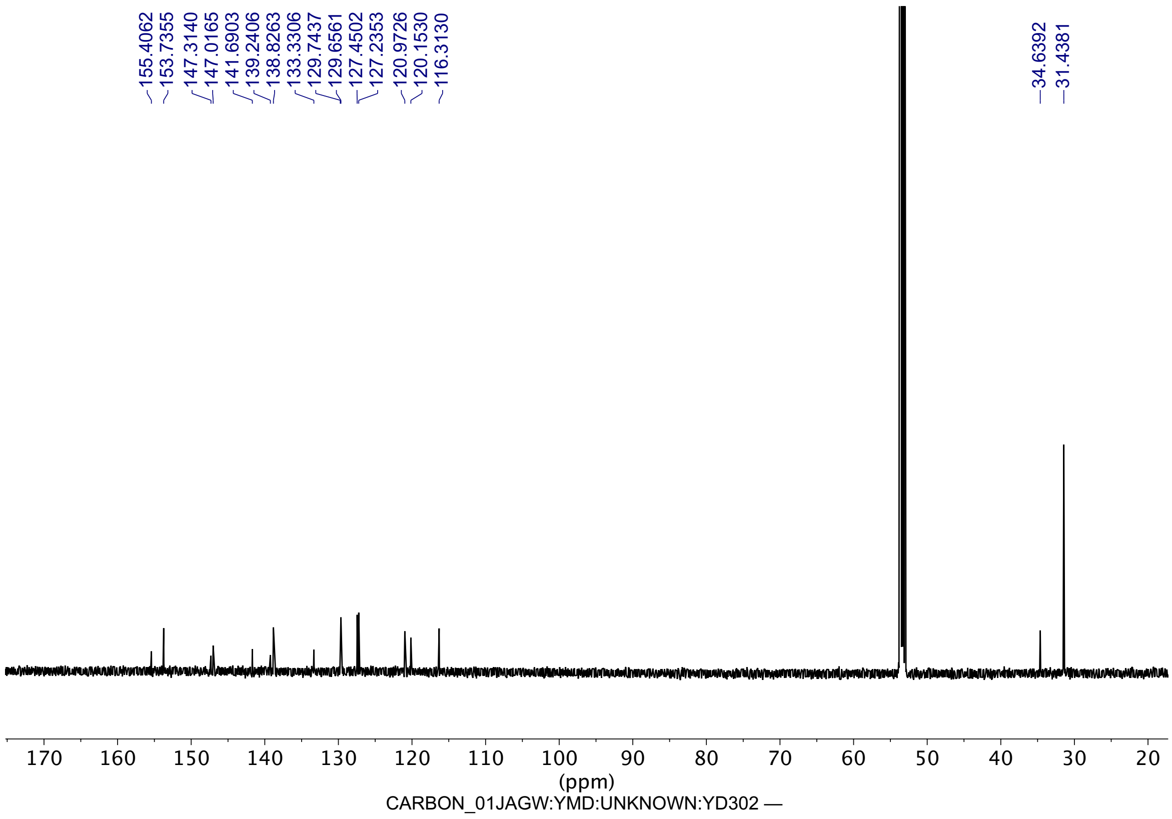

Figure S24. *^{13}C NMR spectrum of PtL^{3} in CD_2Cl_2*

Section 3. X-ray crystallography: crystal data and structure refinements

Table S1 Crystal data and structure refinement for PtL^1, PtL^2 and PtL^3.

	**PtL^1**	**PtL^2**	**PtL^3**
CCDC No.	2484707	2484711	2484706
Empirical formula	$C_{30}H_{30}N_2Pt$	$C_{34}H_{32}N_2Pt$	$C_{38}H_{34}N_2Pt$
Formula weight	613.65	663.70	713.785
Temperature/K	120.00	120.00	120.00
Crystal system	monoclinic	triclinic	triclinic
Space group	$P2_1/n$	P-1	P-1
a/Å	14.1941(5)	13.2316(3)	7.9225(11)
b/Å	10.9744(4)	13.7483(4)	13.1739(18)
c/Å	15.9505(5)	15.6441(4)	13.6608(19)
α/°	90	68.3692(10)	87.845(4)
β/°	107.5540(10)	87.8420(10)	89.704(4)
γ/°	90	85.1322(11)	84.084(4)
Volume/$Å^3$	2368.93(14)	2635.84(12)	1417.2(3)
Z	4	4	2
$\rho_{calc}g/cm^3$	1.721	1.672	1.673
μ/mm^{-1}	5.944	5.349	4.962
F(000)	1208.0	1312.0	706.0
Crystal size/mm^3	0.168 × 0.166 × 0.036	0.09 × 0.05 × 0.01	0.146 × 0.032 × 0.027
Radiation	MoKα (λ = 0.71073)	Mo Kα (λ = 0.71073)	Mo Kα (λ = 0.71073)
2Θ range for data collection/°	4.578 to 59.236	4.156 to 55	4.22 to 55
Index ranges	-19 ≤ h ≤ 19, -15 ≤ k ≤ 15, -22 ≤ l ≤ 22	-17 ≤ h ≤ 17, -17 ≤ k ≤ 17, -20 ≤ l ≤ 20	-11 ≤ h ≤ 11, -18 ≤ k ≤ 18, -19 ≤ l ≤ 19
Reflections collected	58520	67710	36889
Independent reflections	6658 [R_{int} = 0.0473, R_{sigma} = 0.0280]	12091 [R_{int} = 0.0627, R_{sigma} = 0.0472]	6507 [R_{int} = 0.0687, R_{sigma} = 0.0588]
Data/restraints/parameters	6658/0/304	12091/57/709	6507/842/417
Goodness-of-fit on F^2	1.073	1.106	1.045
Final R indexes [I>=2σ (I)]	R_1 = 0.0239, wR_2 = 0.0437	R_1 = 0.0477, wR_2 = 0.0923	R_1 = 0.0692, wR_2 = 0.1642
Final R indexes [all data]	R_1 = 0.0331, wR_2 = 0.0462	R_1 = 0.0629, wR_2 = 0.0972	R_1 = 0.0746, wR_2 = 0.1682
Largest diff. peak/hole / e $Å^{-3}$	0.76/-0.91	5.74/-2.67	6.47/-5.37

Table S2 Crystal data and structure refinement for PtL^0Cl_2, PtL^2Cl_2 and PtL^3Cl_2.

	PtL^0Cl_2	PtL^2Cl_2	PtL^3Cl_2
CCDC No.	2484709	2484712	2484710
Empirical formula	$C_{22}H_{14}Cl_2N_2Pt$	$C_{34}H_{32}Cl_2N_2Pt$	$C_{40}H_{38}Cl_6N_2Pt$
Formula weight	572.34	734.60	954.51
Temperature/K	120.0	120.00	120.00
Crystal system	orthorhombic	orthorhombic	triclinic
Space group	$P2_12_12_1$	$P2_12_12_1$	P-1
a/Å	8.2704(2)	12.1298(5)	9.8883(3)
b/Å	14.7677(4)	13.2304(6)	13.6503(4)
c/Å	14.9882(4)	17.6483(8)	14.8785(4)
α/°	90	90	70.6418(11)
β/°	90	90	81.2945(11)
γ/°	90	90	79.5201(11)
Volume/Å^3	1830.58(8)	2832.2(2)	1854.13(9)
Z	4	4	2
ρ_{calc}g/cm^3	2.077	1.723	1.710
μ/mm^{-1}	7.966	5.170	4.249
F(000)	1088.0	1448.0	944.0
Crystal size/mm^3	0.22 × 0.15 × 0.005	0.18 × 0.14 × 0.06	0.14 × 0.06 × 0.03
Radiation	Mo Kα (λ = 0.71073)	MoKα (λ = 0.71073)	MoKα (λ = 0.71073)
2Θ range for data collection/°	3.872 to 59.988	3.848 to 64.998	4.21 to 63.998
Index ranges	$-11 \le h \le 11$, $-20 \le k \le 20$, $-21 \le l \le 21$	$-18 \le h \le 17$, $-20 \le k \le 20$, $-26 \le l \le 26$	$-14 \le h \le 14$, $-19 \le k \le 20$, $-22 \le l \le 22$
Reflections collected	32961	119186	76877
Independent reflections	5342 [R_{int} = 0.0525, R_{sigma} = 0.0360]	10246 [R_{int} = 0.0529, R_{sigma} = 0.0251]	12887 [R_{int} = 0.0392, R_{sigma} = 0.0278]
Data/restraints/parameters	5342/0/244	10246/0/358	12887/166/488
Goodness-of-fit on F^2	1.052	1.138	1.032
Final R indexes [I>=2σ (I)]	R_1 = 0.0237, wR_2 = 0.0448	R_1 = 0.0446, wR_2 = 0.1041	R_1 = 0.0209, wR_2 = 0.0445
Final R indexes [all data]	R_1 = 0.0275, wR_2 = 0.0461	R_1 = 0.0475, wR_2 = 0.1057	R_1 = 0.0241, wR_2 = 0.0456
Largest diff. peak/hole / e Å^{-3}	1.02/-1.03	7.73/-2.98	1.30/-1.47
Flack parameter	-0.006(5)	0.014(3)	

Table S3 Crystal data and structure refinement for $Pt_2L^1{}_2Cl_2$.

CCDC No.	2484708
Empirical formula	$C_{61}H_{62}Cl_4N_4Pt_2$
Formula weight	1383.165
Temperature/K	120.00
Crystal system	monoclinic
Space group	$P2_1$
a/Å	10.3330(7)
b/Å	10.3055(7)
c/Å	25.7800(16)
α/°	90
β/°	95.731(2)
γ/°	90
Volume/Å^3	2731.5(3)
Z	2
ρ_{calc}g/cm^3	1.682
μ/mm^{-1}	5.334
F(000)	1357.0
Crystal size/mm^3	0.113 × 0.032 × 0.016
Radiation	Mo Kα (λ = 0.71073)
2Θ range for data collection/°	3.96 to 51.36
Index ranges	$-13 \leq h \leq 13$, $-13 \leq k \leq 13$, $-33 \leq l \leq 33$
Reflections collected	74616
Independent reflections	10382 [R_{int} = 0.1114, R_{sigma} = 0.0852]
Data/restraints/parameters	10382/1201/653
Goodness-of-fit on F^2	1.057
Final R indexes [I>=2σ (I)]	R_1 = 0.0744, wR_2 = 0.1729
Final R indexes [all data]	R_1 = 0.0876, wR_2 = 0.1793
Largest diff. peak/hole / e Å^{-3}	3.77/-5.65
Flack parameter	-0.023(6)

Section 4. Frontier orbitals and spin density plots

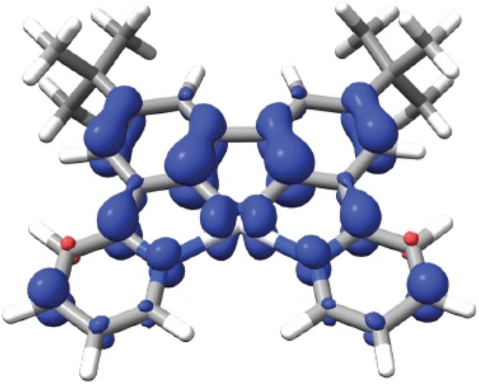

Figure S25. *Spin density plot for the T_1 state of PtL^1.*

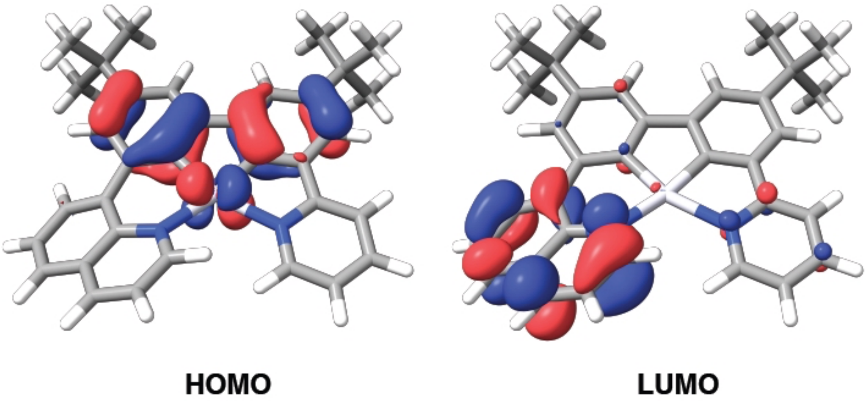

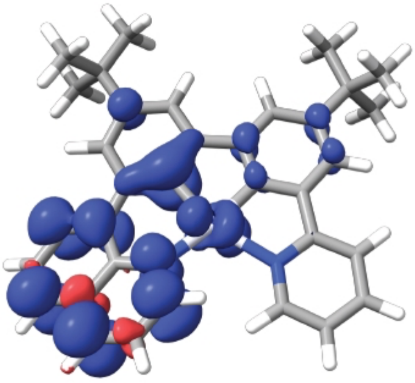

Figure S26. *Frontier orbital plots for PtL^2 at the S_0 geometry (top), and spin density plot of the T_1 state (bottom).*

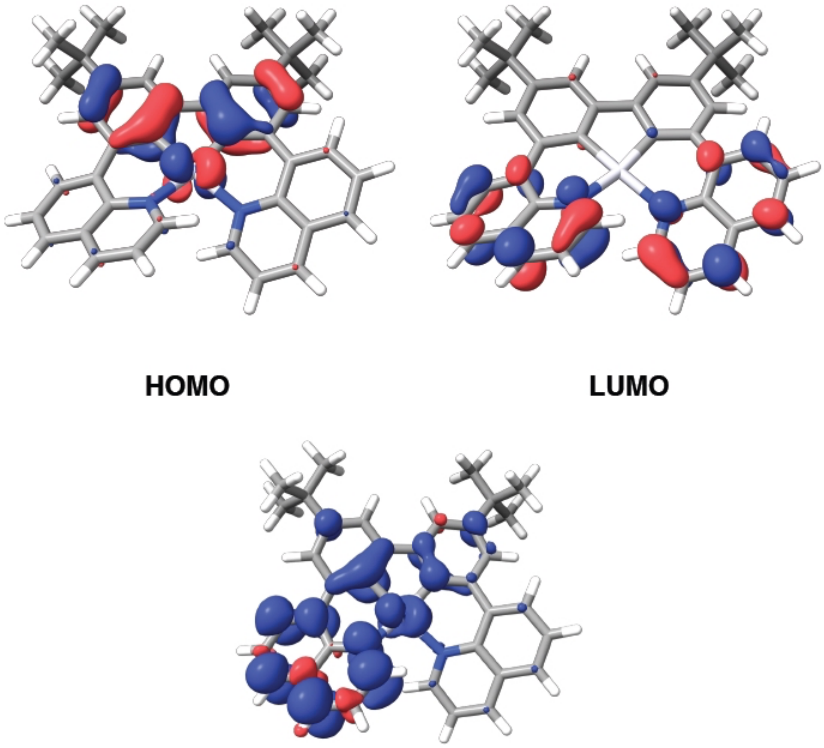

Figure S27. *Frontier orbital plots for PtL^3 at the S_0 geometry (top), and spin density plot of the T_1 state (bottom).*

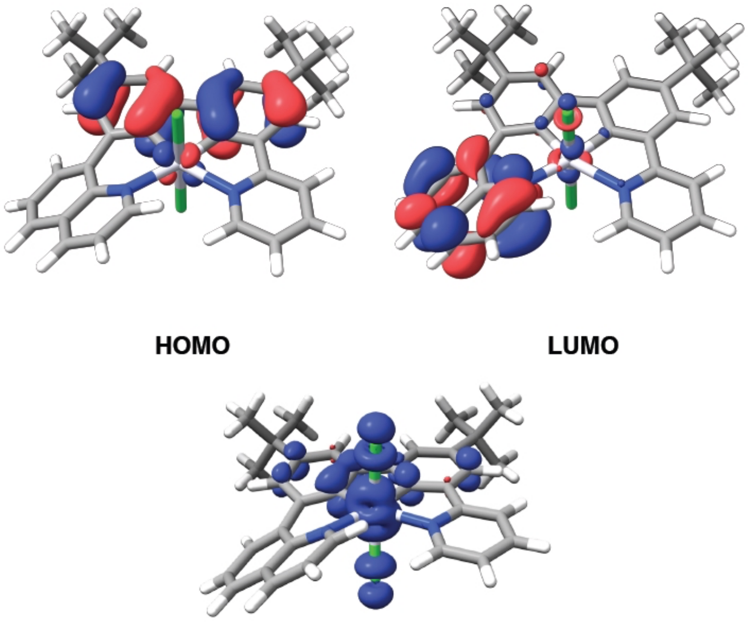

Figure S28. *Frontier orbital plots for PtL^2Cl_2 at the S_0 geometry (top), and spin density plot of the T_1 state (bottom).*

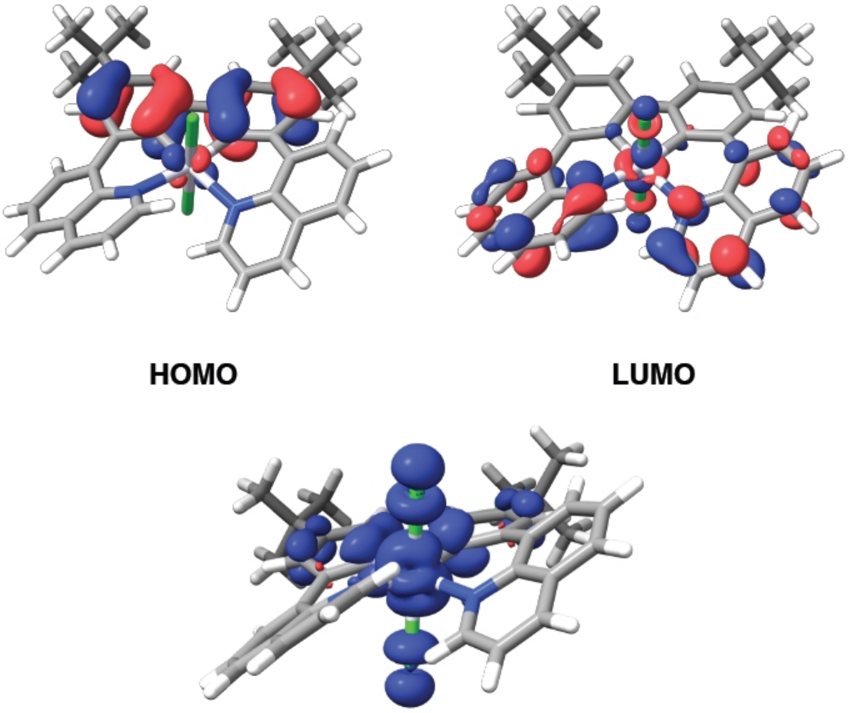

Figure S29. *Frontier orbital plots for* PtL^3Cl_2 *at the* S_0 *geometry (top), and spin density plot of the* T_1 *state (bottom).*

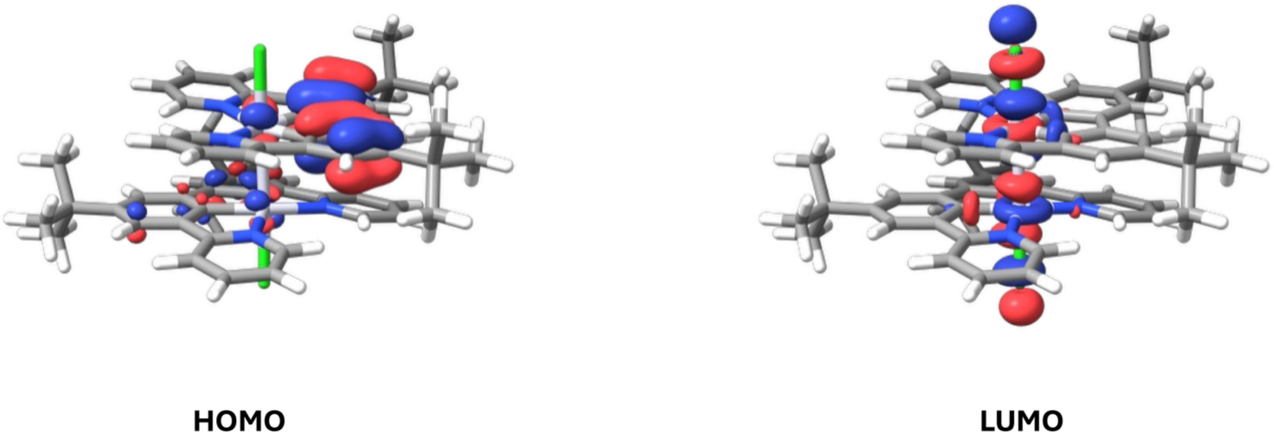

Figure S30. *Frontier orbital plots for* $Pt_2L^1{}_2Cl_2$ *at the* S_0 *geometry*

Table S4 *Population analysis (% contribution) of the frontier orbitals indicated.*

PtL[1]

	HOMO	LUMO	LUMO + 1	LUMO + 2
Pt	21.9	6.8	2.6	3.9
py1	1.2	31.2	21.6	37.4
py2	1.2	31.2	21.3	37.7
ph1	35.3	13	24.1	8.7
ph2	35.4	13.3	24.3	8.9

PtL[2]

	HOMO	LUMO	LUMO+1	LUMO+2
Pt	23.3	2.3	5.3	2.8
quin	3.5	78.9	10.4	4.2
py	1.4	5.8	59	36.9
ph1	34.3	4.6	1.8	22.3
ph2	31.9	3	19	27.1

PtL[3]

	HOMO	LUMO	LUMO+1	LUMO+2
Pt	25	1.7	2.8	5.2
quin1	4	42.4	43.3	8.9
quin2	4.6	43.2	43.5	8.5
ph1	30.2	3.9	1.9	34.4
ph2	30.2	3.3	2.1	34.4

continues

PtL^2Cl_2

	HOMO	LUMO	LUMO+1	LUMO+2
Pt	4.6	4.4	35.8	0.8
quin	-0.1	79.6	6	3.8
py	0	1.1	1.2	57.4
ph1	46.5	4.5	10.2	2.6
ph2	42.4	1.6	5.1	30
Cl1	0.1	0.8	19.5	0
Cl2	0	2.2	17.3	0.2

PtL^3Cl_2

	HOMO	LUMO	LUMO + 1	LUMO + 2
Pt	4.7	9.2	0.1	31.5
quin1	0	37	42.8	9
quin2	0.2	37.5	41.8	8.8
ph1	44.1	1.8	4.7	7.3
ph2	44.1	1.4	4.5	6.9
Cl1	0	3.7	0.1	16.8
Cl2	0.1	3.5	0.1	16.5

continues

$Pt_2L^1{}_2Cl_2$

	HOMO	LUMO	LUMO + 1	LUMO + 2
Pt1	8.2	30.2	1.4	0
Pt2	3.3	30.6	1.4	0.2
Cl1	0	11.7	1	0
Cl2	0	11.8	1	0
L1py1	0	0.1	17.3	25.6
L1py2	0.2	0.2	9.5	3.9
L1ph1	35.5	3.1	5	5.3
L1ph2	30	2.1	6.4	18.2
L2py1	0.3	0.3	11.8	1.8
L2py2	0	0.1	21.1	18.2
L2ph1	8.4	2.8	6.7	3.4
L2ph2	6.1	2.1	9.6	13.7

Section 5. Non-radiative decay of Pt(II) complexes versus excited-state energy

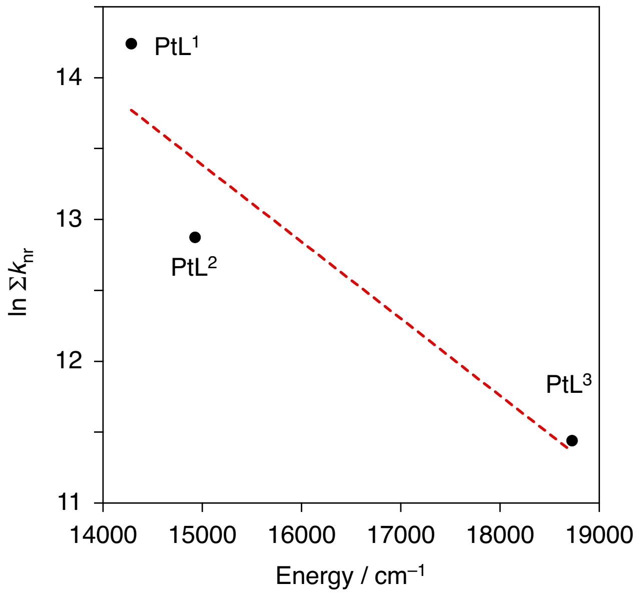

Figure S31. *Plot of ln $\sum k_{nr}$ versus the energy of the emitting state (as estimated from λ_{max}) for PtL^{1-3} at 295 K. The red line shows the best linear fit (but note that a truly linear relationship would not be anticipated anyway, given the structural change from pyridine to quinoline).*
